# Long‐term trends in parasite diversity and infection levels: approaches and patterns

**DOI:** 10.1002/brv.70119

**Published:** 2025-12-19

**Authors:** Cyril Hammoud, Juan Antonio Balbuena, Isabel Blasco‐Costa, Katie O'Dwyer, Rachel A. Paterson, Tomáš Scholz, Christian Selbach, Bernd Sures, David W. Thieltges

**Affiliations:** ^1^ Department of Coastal Systems Royal Netherlands Institute for Sea Research PO Box 59 Den Burg 1790AB The Netherlands; ^2^ Cavanilles Institute of Biodiversity and Evolutionary Biology, University of Valencia PO Box 22085 Valencia 46071 Spain; ^3^ Department of Invertebrates Natural History Museum of Geneva PO Box 6434 Geneva 1211 Switzerland; ^4^ Marine and Freshwater Research Centre, Atlantic Technological University Galway H91 T8NW Ireland; ^5^ Norwegian Institute for Nature Research PO Box 5685 Trondheim 7485 Norway; ^6^ Institute of Parasitology, Biology Centre of the Czech Academy of Sciences České Budějovice 37005 Czech Republic; ^7^ Department of Arctic and Marine Biology UiT – The Arctic University of Norway Tromsø 9037 Norway; ^8^ Water Research Group, Unit for Environmental Sciences and Management North‐West University Potchefstroom 2520 South Africa; ^9^ Aquatic Ecology and Centre for Water and Environmental Research, University of Duisburg‐Essen Universitätsstraße 5 Essen D‐45141 Germany; ^10^ Research Center One Health Ruhr, Research Alliance Ruhr, University Duisburg‐Essen Universitätsstraße 5 Essen D‐45141 Germany; ^11^ Groningen Institute for Evolutionary Life‐Sciences (GELIFES) University of Groningen Groningen AG 9747 The Netherlands

**Keywords:** biodiversity, anthropogenic change, field sampling, historical ecology, Anthropocene

## Abstract

Parasites exist in every ecosystem, affecting nearly all organisms and playing a complex role in human societies. On the one hand, they contribute substantially to biodiversity and support ecosystem stability by performing essential ecological functions. On the other, they can impose health burdens on their hosts, causing diseases in both animals and humans. Despite their significance, our understanding of how parasitic organisms are affected by human‐driven environmental change remains poor. In other well‐studied groups such as free‐living birds, mammals and insects, long‐term ecological data sets have been instrumental in elucidating temporal trends in abundance or diversity and linking them to anthropogenic drivers. For parasites however, overarching long‐term trends in infection levels or diversity have yet to be identified. Here we provide an overview of the research approaches developed to study long‐term changes in parasite systems and the trends highlighted by these studies. Our aims were to help researchers make informed methodological decisions when designing their research, and to provide recommendations for future long‐term research on parasite ecology. To this end, we performed a systematic literature search on long‐term analyses of eukaryotic parasites of wild animals and identified four types of approaches deployed to gather long‐term data: (*i*) long‐term monitoring; (*ii*) snapshot resampling; (*iii*) literature‐based research; and (*iv*) natural history collection‐based studies. Our results revealed striking differences in the temporal scope, geographical scale of sampling, sample sizes and taxonomic resolution of parasite identification among these approaches. However, no overarching trends in parasite infection levels or diversity were identified. When detected, significant temporal changes were often linked to anthropogenic disturbances, but these claims were rarely supported by inferential analyses. Overall, our results show that our understanding of long‐term trends in parasite systems remains hampered by data scarcity and research biases. To address these issues, we advocate for the establishment of large‐scale parasite monitoring programmes combined with existing ecological monitoring projects, as well as the development of new scalable biomonitoring tools. We also highlight the importance of valorising historical data and preserved biological material in museum collections to obtain baseline information on parasite systems.

## I. INTRODUCTION

Human‐mediated environmental changes jeopardise the stability of ecosystems globally (Western, 2001; Hautier *et al*., 2015; Newbold *et al*., 2019), but our understanding of how such disturbances affect organisms differs greatly among taxonomic and functional groups (Collen *et al*., 2009; Pereira, Navarro & Martins, 2012). Despite their ubiquity and overwhelming diversity (Dobson *et al*., 2008), parasites have often been ignored in biodiversity assessments and discussions linked to the current biodiversity crisis (Nichols & Gómez, 2011; Gómez & Nichols, 2013; Dougherty *et al*., 2016). Nevertheless, understanding the long‐term effects of anthropogenic disturbances on parasitic organisms should be a top priority in ecological research. Indeed, human‐mediated environmental changes may alter the distribution of parasites, enhance their transmission dynamics, and increase the frequency of disease outbreaks, with cascading consequences for both humans, domestic animals, and wildlife (Brearley *et al*., 2013; Short, Caminade & Thomas, 2017; Carlson *et al*., 2017; Wells & Flynn, 2022; Sures *et al*., 2023; Deure *et al*., 2024). As importantly, despite their recurring depiction as ecological villains, parasites perform key services in their ecosystems by regulating host populations, mediating inter‐ and intraspecific interactions, and enhancing energy flow through food webs (Kuris *et al*., 2008; Dunne *et al*., 2013; Wood & Johnson, 2015). Given their overwhelming diversity and important ecological roles of parasites in ecosystems, parasite ecologists have advocated for the development of a coordinated agenda for parasite conservation (Gómez, Nichols & Perkins, 2012; Dougherty *et al*., 2016; Carlson *et al*., 2020), which has recently led to the creation of the International Union for Conservation of Nature (IUCN) Species Survival Commission (SSC) Parasite Specialist Group, aiming to assess the conservation status of parasite species within the *Red List* framework (Hopkins & Kwak, 2023). As pointed out by these authors, parasite conservation requires greater knowledge of temporal trends in parasite infection levels and diversity than is currently available, and a better understanding of the effects of anthropogenic environmental changes on parasites (Gómez & Nichols, 2013; Carlson *et al*., 2020).

Long‐term data are essential to characterise trajectories of ecological assemblages (Magurran *et al*., 2010). Therefore, long‐term ecological data sets have been, and continue to be, instrumental in identifying and characterising the current anthropogenic biodiversity crisis (Ducklow, Doney & Steinberg, 2009; Hendershot *et al*., 2020; Montràs‐Janer *et al*., 2024). Just as in free‐living (i.e. non‐parasitic) organisms, long‐term data on parasites may be derived from a variety of sources, including field observations or specimens preserved in natural history collections. However, highly resolved long‐term information on changes in natural ecological populations or assemblages is typically only available for groups that have been both intensively studied and well‐preserved in natural history collections (Wood *et al*., 2023a). Based on these long‐term data sets, declines and shifts in bird (Rosenberg *et al*., 2019), mammal (Hoffmann *et al*., 2011) and insect (Klink *et al*., 2020) diversity have been documented. Parasites, however, are typically understudied (Gómez & Nichols, 2013; Dougherty *et al*., 2016), especially if they neither threaten humans nor domesticated animals or plants, and consequently, are poorly represented in natural history collections (Harmon, Littlewood & Wood, 2019). In addition to the lack of attention to parasites of wildlife, studying these organisms is laborious as only a fraction of their host population is typically infected (Shaw, Grenfell & Dobson, 1998), thus increasing the effort needed to attain acceptable sample sizes. Moreover, intrusive and potentially lethal examination of individual hosts is often required to diagnose infections, which can be particularly problematic if they are of conservation concern. Additionally, the taxonomy of many parasite groups is notoriously complex and incomplete, which further hampers their study (Brooks & Hoberg, 2001; Poulin, 2014; Poulin & Presswell, 2022; Scholz, 2024).

Nonetheless, gathering long‐term data on parasites remains essential for unlocking key knowledge on how parasite infection levels and diversity have changed in the past, and to predict future trajectories. Despite the challenges highlighted above, for decades researchers have deployed efforts and ingenuity to gather such knowledge, making use of field observations, historical data and natural history collections. In this study, we conduct a systematic review of the literature to: (*i*) critically evaluate the strengths of different approaches used to characterise long‐term changes in parasite diversity and infection levels, and (*ii*) highlight potential caveats for future work. Our review also provides a qualitative assessment of the long‐term trends reported so far and their connections with anthropogenic drivers. Ultimately, we expect the work presented herein will support researchers in making informed methodological decisions when designing their research and provide recommendations for the direction of future long‐term research in parasite ecology.

## II. MATERIALS AND METHODS

### (1) Systematic literature search

To provide an accurate and comprehensive overview of the literature on long‐term trends in parasite infection levels and diversity, we compiled an exhaustive bibliographic data set using a systematic approach. We searched for peer‐reviewed long‐term studies of parasites in the *Scopus*, *Web of Science* and *Pubmed* databases using the following query (accessed on 5th July 2024): ALL(‘*parasite’ OR ‘*parasitic’) AND ALL(‘*diversity’ OR ‘abundance’ OR ‘species richness’ OR ‘prevalence’) AND ALL(‘temporal trend*’ OR ‘temporal change*’ OR ‘temporal turnover*’ OR ‘temporal dynamic*’ OR ‘temporal variation*’ OR ‘long‐term trend*’ OR ‘long‐term change*’ OR ‘long‐term turnover*’ OR ‘long‐term dynamic*’ OR ‘long‐term variation*’) AND NOT ALL(‘livestock’ OR ‘cattle’ OR ‘veterinar*’ OR ‘medical*’ OR ‘plant*’). We exported all references as RIS files, compiled them into one bibliography using the *ZOTERO* manager (Takats *et al*., 2023), and removed duplicate studies. Finally, we screened the abstracts and methodological sections of each study and selected only those fitting the following criteria: (*i*) peer‐reviewed ‘long‐term’ studies, which we defined as spanning a period greater than or equal to 5 years. This threshold is admittedly lower than the 10 years sometimes proposed as a minimum to detect population trends in vertebrates (White, 2019). However, the generation time of most parasites is much shorter than that of vertebrates (Kochin, Bull & Antia, 2010) meaning that the effect of environmental changes on their populations might be detected faster (Strayer *et al*., 1986). Furthermore, focusing on studies spanning at least 5 years excludes short longitudinal studies focused on seasonal dynamics. (*ii*) Studies containing observational field data on parasite infection levels (a term used throughout this article to denote any quantitative measures of parasite infections in host populations such as prevalence, mean abundance or mean intensity of infection) or diversity (from species richness to more complex measures of ecological diversity). (*iii*) Studies dealing with eukaryotic parasites of wild or feral metazoan hosts. To be as exhaustive as possible, we also performed backward reference searches and assessed the validity of potentially relevant studies that did not appear in the systematic search but were encountered through the backward search. Our search in the literature databases returned a total of 1580 studies (694 from *Scopus*, 621 from *Web of Science* and 265 from *Pubmed*; see online Supporting Information, Fig. S1). A total of 640 duplicates were removed, and 144 potentially relevant studies that were not located by this database search were identified by backward searches from the remaining 940 studies. From these 1084 studies, 244 met our selection criteria and were incorporated in our final bibliographical data set (Table S1, also available at https://doi.org/10.5281/zenodo.17856798). From the 840 studies that did not fit our selection criteria, 317 were excluded because they treated an unrelated topic or did not incorporate field data, 295 spanned over a duration less than 5 years, 180 focused on hosts that were not wild or feral metazoans, and 48 studies focused on prokaryotic parasites (Fig. S1).

### (2) Long‐term research approaches

Based on a first basic assessment of the literature gathered and our knowledge of long‐term research in parasite ecology, we identified four research approaches: (*i*) long‐term monitoring, defined as the continuous collection of data from the field over the study period; (*ii*) snapshot resampling, corresponding to the re‐characterisation, after some time, of a parasite system that had baseline information available; (*iii*) literature data‐based studies, which rely on published data to characterise long‐term changes; (*iv*) natural history collection‐based studies, which use archived preserved material (typically host specimens) to investigate changes in parasite populations or assemblages. Each study in our data set was categorised into one of these predefined categories of research approaches.

**Fig. 1:**
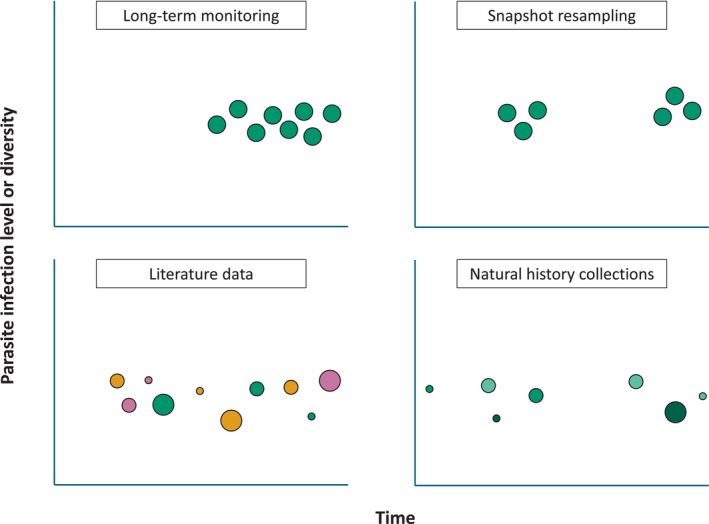
Schematic representation of four research approaches to study long‐term variation in parasite infection levels or diversity. Each dot represents a hypothetical parasite or host sample at a certain time (from left to right, ancient to recent), with size proportional to sample size and colour‐consistency informing the geographical scale of the samples: dots with greater differences in colours signal samples collected from more distant locations.

As illustrated in Fig. 1, we hypothesised that these categories may differ in terms of temporal span and coverage, geographical scope, and sample sizes. Based on prior planning and continuous research effort, long‐term monitoring studies were expected to yield regular and temporally comprehensive samples with large sample sizes. However, these efforts were anticipated to focus on narrow geographical scales and cover short temporal spans due to the high, continuous resource investment required. We expected snapshot resampling studies also to yield large sample sizes and focus on narrow geographical scales, whilst potentially covering longer time spans than monitoring studies as they do not require similarly high continuous resource investment. By contrast, we hypothesised that literature‐based research would result in the agglomeration of sample points from distant locations, thereby broadening the geographical scope. By leveraging published historical records, we expected these studies to cover longer temporal spans than monitoring or snapshot resampling projects, while relying on the large, combined sample sizes of these records. Finally, we anticipated that natural history collection‐based studies would provide the longest temporal insights, due to the use of century‐old, preserved host specimens. However, we expected these studies to consist of samples from moderately distant locations and to rely on low sample sizes due to the limited availability of preserved material.

### (3) Methodological information

From each screened study, we recorded the beginning and end of the study year, total duration (range between the collection date of first and last sample), broad geographical scope (local, regional, national, continental or global), study system coordinates (approximate centroid), type of environment studied (terrestrial, marine, freshwater, brackish or a combination thereof), host(s) (fish, mammals, birds, molluscs, amphibians, crustaceans, reptiles, insects, or multiple groups together), and parasite groups (helminths, arthropods, protists, fungi, myxozoans, or leeches) studied, host sample size (total number of host specimens inspected for parasite infections in the study), and parasite identification method (morphological and/or molecular). We also investigated the accuracy of parasite identification as the number of parasite taxa that could be identified at species, genus, or higher taxonomic level. Additionally, we categorised the general aim of each study as characterising: (*i*) changes in parasite infection levels; (*ii*) changes in parasite diversity (including changes the richness and/or composition of parasite species assemblages); (*iii*) changes in the distribution patterns of parasites; (*iv*) the general diversity of parasites in an ecosystem; or (*v*) the impact of parasites on their hosts. We also recorded the type of significant temporal changes, if any, identified by the authors in the host–parasite system analysed. More specifically, we determined whether authors reported changes in parasite infection levels, alpha diversity, or in the composition of parasite species assemblages (shifts in presence–absence patterns), as well as the direction of these changes where relevant. Finally, when significant temporal trends were reported, we recorded whether authors linked them to anthropogenic environmental changes, the nature of these changes, and whether these links were supported by inferential statistical analyses.

### (4) Statistical analyses

We first investigated whether the long‐term research approaches implemented differed in terms of temporal span by using pairwise Wilcoxon rank sum tests with Benjamini–Hochberg corrections to perform pairwise comparisons of the mean time span (T1). We also explored whether studies within the four predefined research categories tend to focus on different geographical scales as hypothesised above (Fig. 1). To accomplish this, we first composed a contingency table containing the number of studies focused on either local, regional, national, continental, and global scales grouped by approach. Then, we performed a chi‐squared test to evaluate whether the distribution of studies among these categories was independent from the research approach (T2). We applied a similar chi‐squared based approach to investigate whether studies within research approach categories tend to focus on distinct geographical scales (T3) or types of environments (T4), and additionally to assess potential differences among research approach categories in host groups (T5), resolution of the taxonomic identification of parasites (species, genus, or family or higher, T6), and general research aims (T7). Additionally, we used a chi‐squared test to investigate whether the resolution of the taxonomic identification of parasites (identical categories as in T6) depends on the identification method used (morphological, molecular, or both, T8). We also aimed at providing an estimate of the sample sizes supported by each research approach. To achieve this, we first computed the ratio between the host sample size of the study and its duration (number of hosts per year) to produce a time‐adjusted metric of sample size. Then, we compared the means of these adjusted sample sizes among all four approaches using pairwise Wilcoxon rank sum tests with Benjamini–Hochberg corrections (T9). A summary of the statistical tests performed is provided in Table S2. All statistical analyses were run in *R* 4.3.1 (R Core Team, 2023) with significance levels fixed at α = 0.05 when applicable. The related R script is available on Zenodo (https://doi.org/10.5281/zenodo.17856798).

## III. RESULTS

### (1) Methodological patterns

**Fig. 2:**
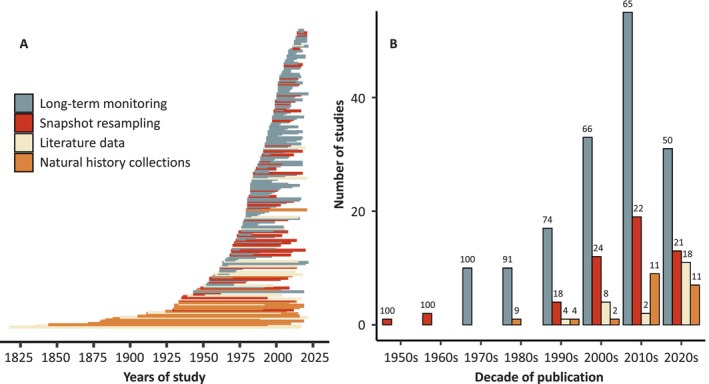
(A) Temporal span of studies according to research approach, ordered by year of commencement (each line represents one publication, ranging from the year of the oldest sample studied to the most recent). (B) Number of studies published each decade since the 1950s, coloured by research approach implemented. The value above each bar represents the proportion (in per cent) of studies in the given research approach and decade.

**Fig. 3:**
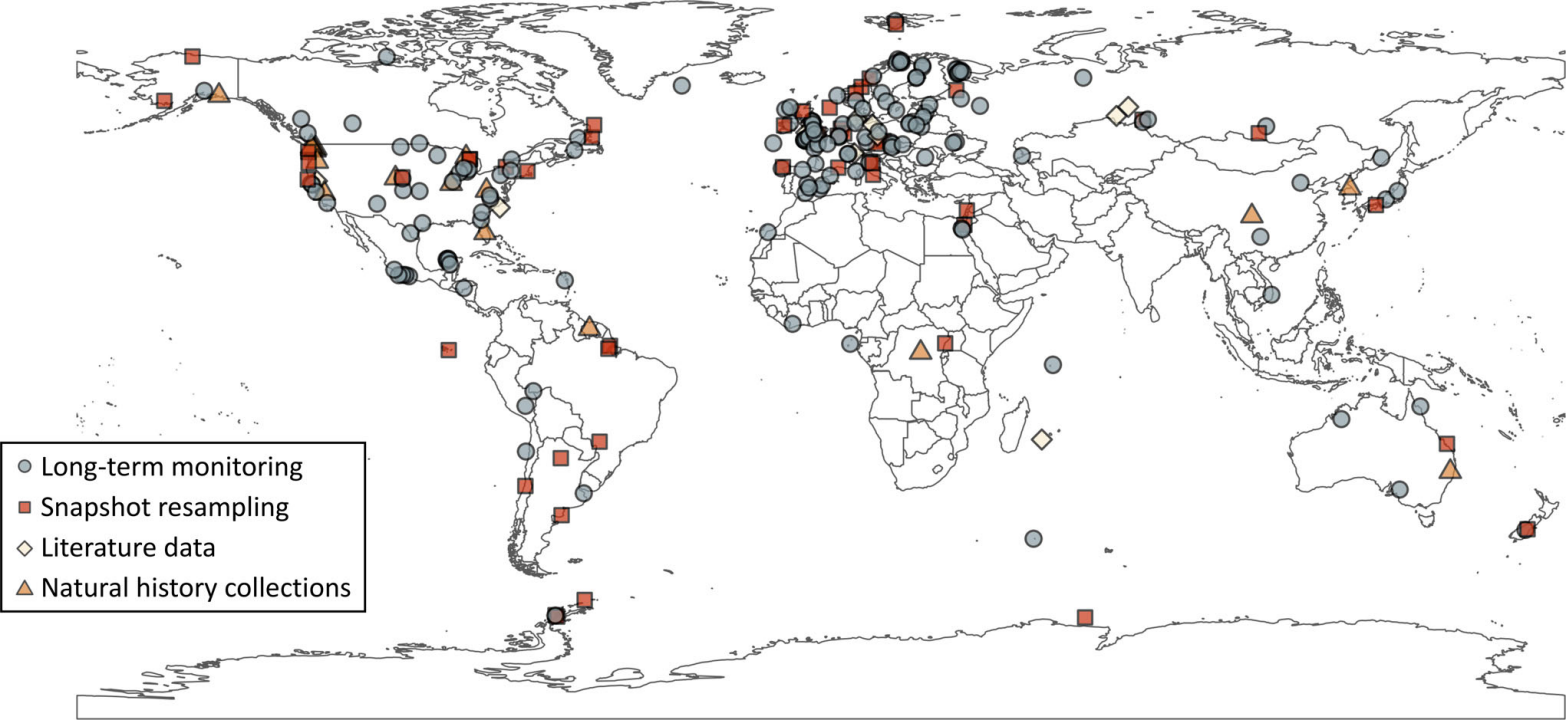
Map of the geographical location of 236 studies included in the bibliographical data set (geographic coordinates are the approximate centroid of the study area, the eight studies focused on global patterns are not represented). Points are coloured by approach and slightly jittered to avoid excessive overlap. Maps focusing on Europe and North America are presented in Fig. S3.

Long‐term monitoring was adopted by most studies in our bibliographic data set (*N* = 156) to characterise changes in parasite systems, followed by snapshot resampling (*N* = 51), natural history collection‐based studies (*N* = 19) and literature‐based studies (*N* = 18). The number of long‐term monitoring studies published has increased through the decades, starting with one study in the 1950s and peaking at 85 publications during the 2010s (Fig. 2). Additionally, the research approach used seems to have shifted over time: in the 1970s and 1980s, most studies (>90%) focused on long‐term monitoring, whereas this proportion gradually declined over time to ~50% in the 2020s. This decrease was concomitant with an increase in snapshot resampling studies (rare before the 1980s, and increasing to a stable ~20% in the 2000s and thereafter), and more recent increases in the representation of natural history collection‐based research (reaching >10% in the 2010s and 2020s) and literature‐based studies (most common in the 2020s). Research duration ranged from five to 200 years (mean ± SD: 28.4 ± 31.2 years) and differed significantly among research approaches (Table S2). Long‐term monitoring research tended to cover shorter time spans (16.0 ± 11.4 years) than snapshot resampling (27.2 ± 18.6 years), literature (70.6 ± 51.3 years) and natural history collection‐based studies (94.3 ± 30.0 years, Figs 2A and S2A). The studied systems were predominantly located in Europe (*N* = 111), followed by North America (*N* = 70), Asia (*N* = 20), South America (*N* = 13), Africa (*N* = 9), Oceania (*N* = 7) and Antarctica (*N* = 6) (Fig. 3, see Fig. S3 for detailed results from Europe and North America). The remaining eight studies covered a substantial portion of the globe and were therefore not assigned to a specific continent. The geographical scope of research was significantly linked to the research approach (Table S2), with local‐scale research well represented in long‐term monitoring and snapshot resampling studies, whereas literature and natural history collection‐based studies were associated with national, continental, and global analyses (Fig. S2B). Similarly, the four research approaches were not equally represented among continents (Table S2). Natural history collection‐based studies are over‐represented in North America and under‐represented in Europe, and snapshot resampling studies are over‐represented in Antarctica and South America (Fig. S4). Finally, ~61% of studies in our data set focused on aquatic study systems (73 in freshwater environments, 61 in marine and 15 in estuarine systems), whereas ~34% focused on terrestrial systems and the remaining ~5% encompassed multiple types of environments. Again, study environments were not represented equally among research approaches (Table S2) as terrestrial systems tended to be well represented in long‐term monitoring studies, and studies including multiple environments tended to be well represented in natural history collection and literature data studies (Fig. S5).

**Fig. 4:**
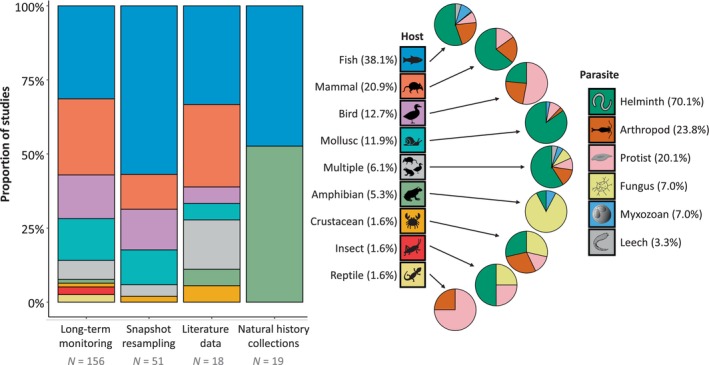
Distribution of host groups depending on the research strategy implemented (left), and pie charts of the proportion of studies investigating different parasite groups for each host (right). Sample size values under the bar plot represent the number of articles in each category. Percentage values in parentheses show the proportion of studies (all strategies included *N* = 244) that focused on the specific host or parasite group. Exact proportions of each parasite group attributed to each host group are presented in Table S3.

Most studies in our bibliographic data set focused on one (58.6%) or two (10.7%) host taxa (typically species but sometimes genera or broader taxonomic units), while a few literature‐based studies included hundreds of host taxa (Fig. S6). Contrastingly, most studies (63.5%) included more than one parasite taxon (sometimes genetic strains but generally species, genera or broader taxonomic units). The number of parasite taxa studied scaled positively with the number of host organs surveyed (Fig. S7). Most studies focused on fish hosts (38.1%), followed by mammals (20.9%), birds (12.7%), molluscs (11.9%), amphibians (5.3%), and finally reptiles, crustaceans, and insects (each representing 1.6% of studies; Fig. 4). The remaining 15 studies (6.1%) included hosts from multiple taxonomic groups. We found significant differences in the representation of hosts among research approaches (Table S2), the most striking pattern being the high representation of amphibians in natural history collection‐based studies and the absence of other host groups except fish in the same category (Fig. 4). The target host group also depended on the studied environment, with mammals and birds encompassing most hosts in terrestrial studies, whereas aquatic studies were overwhelmingly focused on fish and, to a lesser extent, molluscs (Fig. S8). Except for rare exceptions, all studies that included multiple environment types focused on amphibians.

Most studies (82.8%) concerned one broad group of parasites, whereas the remaining encompassed two to five groups. Helminths were the focus of most studies (70.1%), followed by arthropods (23.8%), protists (20.1%), fungi (7.0%), myxozoans (7.0%), and finally leeches (3.3%). Helminths were overwhelmingly present in studies focused on fish, mammals, molluscs, and insects. By contrast, birds were mostly studied for protists, amphibians for fungi, and crustaceans for arthropod parasites, helminths and fungi in equal proportions (Fig. 4, Table S3). The difference in focal parasite groups among studied environments seemed less pronounced than for host groups. Helminths were included in most aquatic studies and in many terrestrial studies (Fig. S9). Protists however were more represented in terrestrial studies, and most studies encompassing multiple environments focused on fungi.

**Fig. 5:**
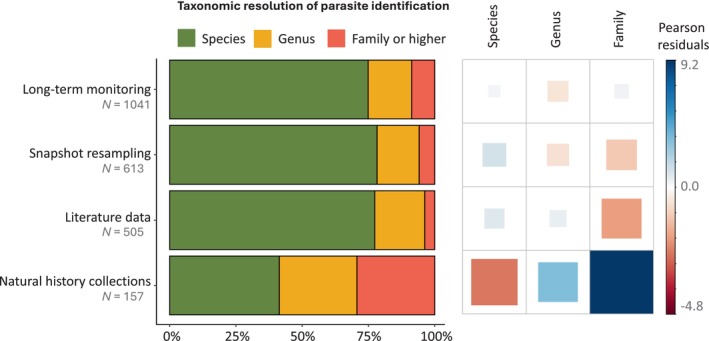
Taxonomic resolution of parasite identification depending on the research approach implemented. Sample size values refer to number of identified parasites across studies. The plot matrix on the right confirms, using the Pearson residuals of the Chi‐squared test, that parasites identified in natural history collection‐based studies are identified less often to species level and more frequently to genus or family level than in other approaches.

Parasite specimens were identified with a degree of precision that varied significantly with the research approach implemented (Table S2). While ~75% of parasites were identified to species level in long‐term monitoring, snapshot resampling and literature‐based research, this proportion dropped to ~40% of parasite taxa in natural history collection‐based studies. Half of the remaining taxa from natural history collection‐based studies were identified to genus level and the other half to family level or higher (Fig. 5). Furthermore, the resolution of parasite identification also depended significantly on the identification method (Table S2). Indeed, a greater proportion of specimens were identified to species level using either molecular tools or a combination of morphological and molecular methods than based on morphology only (Figs S10 and S11).

**Fig. 6:**
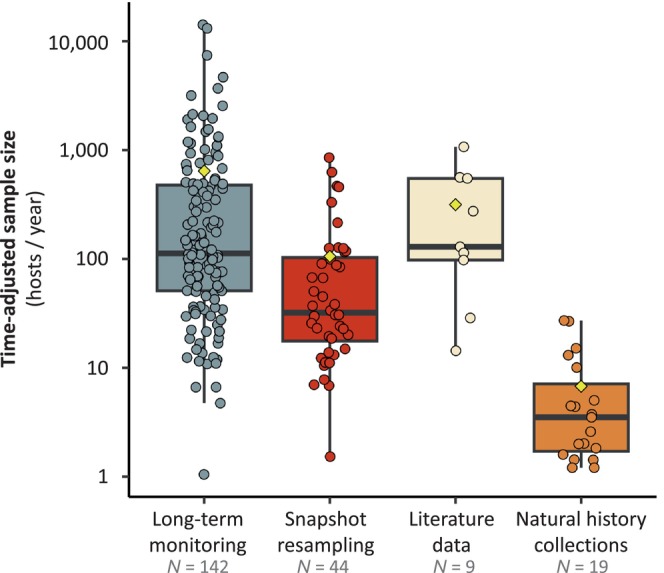
Boxplot of the differences in time‐adjusted study sample size among research approaches. The boxes delimitate the upper and lower quartiles, with the median value represented as a thicker black line (the vertical whiskers outside the boxes indicate the range of data, with points falling outside this range being considered outliers). Points each represent a study and were slightly jittered to improve readability. Yellow diamonds show the group‐specific mean values compared using Wilcoxon rank sum tests. Sample sizes below the four approach categories indicate the number of studies in each category for which this information could be retrieved.

Finally, the average time‐adjusted sample sizes (ratio between the total number of hosts examined in a study and the time span of the study) differed significantly among research approaches, except between long‐term monitoring and literature‐based studies (Fig. 6; Table S2). These two approaches reached similarly high adjusted sample sizes, averaging above 600 hosts/year for long‐term monitoring and 300 hosts/year for literature‐based studies (Fig. 6). Snapshot resampling studies relied on significantly lower sample sizes (average ~100 hosts/year), while the lowest sample sizes were observed in natural history collection‐based studies (~7 hosts/year; Fig. 6).

**Fig. 7:**
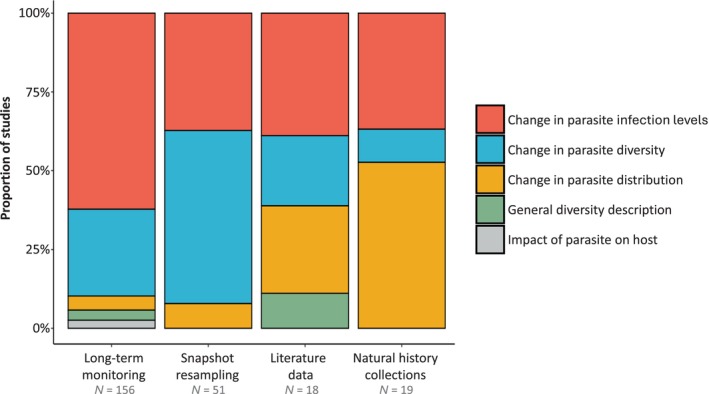
General aim of studies depending on the research approach implemented. Sample sizes (*N*) correspond to the number of studies in each category.

The most frequent study aim outlined by authors across our bibliographic data set was assessing changes in parasite infection levels (53.3%), followed by quantifying changes in parasite diversity (i.e. in the richness and/or composition of parasite species assemblages; 31.6%), characterising changes in parasite distribution patterns (9.8%), providing a general description of parasite diversity (2.9%), and evaluating the impact of parasites on their hosts (1.6%). These aims were not homogeneously represented among research approaches (Table S2). Long‐term monitoring studies tended to focus on changes in the infection levels of parasites and less often on changes in their distribution patterns, whereas snapshot resampling studies most often aimed at evaluating changes in parasite diversity (Fig. 7). Finally, literature‐based studies covered a more balanced mix of research aims, whereas natural history collection‐based studies were overwhelmingly focused on tracking changes in the distribution of parasites and to a lesser extent on changes in parasite infection levels.

### (2) Patterns in long‐term trends

**Fig. 8:**
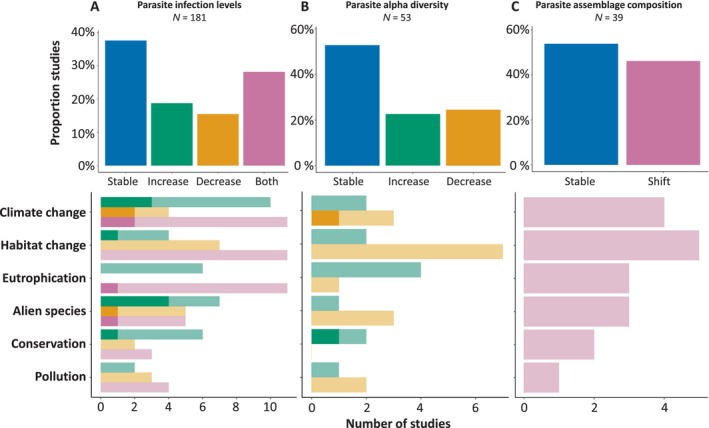
Reported parasite infection levels (A), alpha diversity (B) and assemblage composition (C) (sample size represents the total number of studies reporting on the specific metric). Top panel shows long‐term trends and bottom panel shows number of studies linking specific changes in these parasite metrics to various kinds of anthropogenic changes. Light‐shaded bars indicate the total number of causal links suggested, whereas dark‐shaded bars represent the number of claims supported by inferential analyses.

Changes in parasite infection levels (181 studies, 74.2%) were commonly reported, even when they were not the study's core aim (Fig. 8). Stable parasite infection levels were most commonly observed (37.6%), whereas 18.8% of studies reported increasing infection levels, 15.5% reported decreases, and 28.1% found a combination of stable, increasing and/or decreasing infection levels depending on the parasite species. Six broad anthropogenic drivers were invoked to explain these changes: climate change (13.3% of studies reporting significant infection level changes), habitat change (12.2%), aquatic eutrophication (9.4%), the introduction of alien species (9.4%), conservation measures (6.1%), and pollution (5.0%) (Fig. 8A). Climate change, eutrophication, the introduction of alien species and conservation measures were invoked more often to explain increases in parasite infection levels rather than decreases. Authors linked increased parasite infection levels to these anthropogenic drivers by highlighting one or multiple steps of the parasite life cycle that may benefit from the related environmental or ecosystemic changes. For example, several studies suggested that either climate change, eutrophication, or conservation measures may benefit intermediate hosts or final host populations and thereby increase parasite infection levels (Green *et al*., 2011; Møller *et al*., 2013; Pisano *et al*., 2019; Quinn *et al*., 2021; Mastick *et al*., 2024b). Warming temperatures were also mentioned as potentially increasing host susceptibility towards parasite infections, resulting in increased infection levels (Fiorenza *et al*., 2020). Finally, both the introduction of alien parasite species in systems with native competent hosts and the introduction of alien competent hosts in systems where they could be infected by native parasites were proposed as a mechanism driving increased infection levels of alien and native parasites, respectively (Audenaert *et al*., 2003; Tragust *et al*., 2015). Unlike the anthropogenic changes mentioned above, authors linked habitat changes and pollution more frequently to decreases in parasite infection levels than increases (Fig. 8A). Authors tended to link habitat changes to reduction in host availability (Cort, Hussey & Ameel, 1960; Ceballos *et al*., 2006), whereas pollution was either suggested to have a detrimental impact on the health of hosts (Thomas, 2002) or parasites (Sitko & Heneberg, 2021). However, most connections between anthropogenic impacts and infection levels changes (84.2%) were not supported by inferential analyses but rather were based on *post‐hoc* speculation. For stable (or fluctuating without a strong directional trend) infection levels of parasite species through time, researchers generally linked this pattern to stable environmental conditions (Loy & Haas, 2001), to a high degree of flexibility in the life cycle of the parasite, which may allow it to rely on different hosts depending on the impact of environmental disturbances (Yurlova *et al*., 2006), or to the lack of impact of existing environmental disturbances on the parasite and its hosts (Khan, 2007; Bentley & Burgner, 2011).

Changes in parasite alpha diversity (53 studies, 21.7%) (Fig. 8B) were assessed less often than changes in infection levels. Of these studies, approximately half observed stable parasite alpha diversity, with significant gains and losses similarly likely for the remaining studies (22.6% and 24.5% respectively). Increased parasite diversity was most often linked to aquatic eutrophication and conservation measures, which authors related to higher host availability and diversity (e.g. Huspeni & Lafferty, 2004; Kuzmina *et al*., 2022a). Contrastingly, studies tended to associate reduced alpha diversity to climate change, habitat change, alien species and pollution. Climate change and the introduction of alien species were often suspected to act by directly or indirectly decreasing the availability of key intermediate hosts for specific parasite species present in the ecosystem, therewith disrupting their transmission (e.g. Morley & Lewis, 2006; Désilets *et al*., 2013; Giari *et al*., 2020). Changes in host feeding habits due to anthropogenic habitat changes and waste pollution were also proposed to disrupt parasite transmission, resulting in the local extinction of some species (Sitko & Heneberg, 2020). However, these claims were supported by inferential tests in only two out of 24 studies. Most papers did not provide explanations to justify stable alpha diversity, but some noted that this diversity metric may be resilient to environmental changes (Preisser *et al*., 2022; Syrota *et al*., 2023) and that changes in species assemblages might not necessarily lead to changes in alpha diversity (Preisser *et al*., 2022).

Finally, shifts in parasite assemblages were analysed less often than changes in infection levels and alpha diversity (39 studies, 16.0% of total) (Fig. 8C). Slightly less than half (46.2%) of these studies found significant changes in the composition of parasite assemblages (i.e. addition or loss of parasite species to the assemblages), whereas the rest (53.8%) did not. However, these latter studies sometimes still found changes in the structure of these assemblages, for example driven by variations in parasite infection levels and in patterns of species dominance over time (e.g. Cortés & Muñoz, 2009; Carpio‐Hernández *et al*., 2024). Authors preferentially related shifts to habitat and climate changes and rarely to pollution or conservation measures, but these claims were never based on empirical evidence. Changes in the composition of parasite assemblages were often suspected to be associated with human‐driven changes in the availability of intermediate or final hosts, which eventually determine which parasite species would locally thrive or decline (Kennedy, 1997; Dzikowski, Paperna & Diamant, 2003b; Bauer & Whipps, 2015). Some studies observed temporally stable parasite assemblages (sometimes across multiple generations of hosts; Byers, Holmes & Blakeslee, 2016) which authors sometimes linked to stable host demographics, feeding habits and/or habitat use (Thieltges *et al*., 2008; Byers *et al*., 2016), generally themselves related to environmental stability or rapid return to initial environmental conditions after a disturbance (e.g. Huspeni & Lafferty, 2004; Byers *et al*., 2016). Other studies, however, noted that environmental changes did not systematically lead to compositional changes (e.g. Syrota *et al*., 2023; Carpio‐Hernández *et al*., 2024) or did not discuss the potential links between compositional stability and anthropogenic activities (e.g. Kennedy & Moriarty, 2002; Dezfuli *et al*., 2014).

## IV. DISCUSSION

### (1) Long‐term research in parasite infection levels and diversity

In this review, we summarised the state of research on long‐term investigations of wildlife parasites. This data set from 244 studies is, to our knowledge, the most exhaustive body of literature on long‐term host–parasite systems gathered to date. We identified four different research approaches: Most studies (64%) collected data for long‐term monitoring, i.e. regular sampling of a study system over an extended period, whereas the remaining inferred trends through resampling systems with existing historical information (21%), using specimens preserved in natural history collections (8%) or compiling published data sets (7%). Each of these approaches comes with advantages and disadvantages (Table 1) and reveals distinctive patterns as well as research gaps and biases that can inform future directions for research in the field.

**Table 1:** Advantages and drawbacks of the four main approaches identified during the literature review.

	Advantages	Drawbacks
Long‐term monitoring	High temporal resolution Large sample sizes relative to time span investigated High consistency in diagnostic and identification methods	Highly demanding sampling investment Short time span investigated Few studies started before the 1960s Generally relies on lethal sampling of large numbers of hosts and parasites
Snapshot resampling	Valorisation of existing data High time and cost‐efficiency Wider time span investigated than long‐term monitoring	Poor temporal resolution Dependence on availability and reliability of historical records Potential inconsistencies in sampling, diagnostic and identification methods as well as sampling efforts. Generally relies on lethal sampling of moderate numbers of hosts and parasites
Literature data	Valorisation of existing data No investment in sampling Second longest time span investigated Large sample sizes relative to the time span investigated	Scattered geographical and temporal sampling Dependence on availability, access to, and reliability of historical records Poor consistency in diagnostic and identification methods
Natural history collections	Longest time span investigated Valorisation of existing natural history collections	Geographical and temporal distribution of sampling dependent on specimen availability Specimen preservation and metadata availability may limit usability Low taxonomic resolution in the identification of parasites Potentially destructive for museum specimens Low sample sizes relative to the time span investigated

### (2) Research approach‐specific advantages and caveats

Long‐term monitoring studies represented most of the published material, partly because other approaches were only developed or gained momentum in the last few decades. Long‐term monitoring can contribute immensely to our understanding of changes in parasite diversity as a result of their high temporal resolution and detailed inventories of parasite assemblages. In fact, these studies have shown that the infection levels of some parasites may show strong temporal fluctuations, either cyclic or seemingly stochastic, both at the scale of months and years (Tenora, Wiger & Baruš, 1979; Kennedy, Shears & Shears, 2001; Bensch *et al*., 2007). These patterns suggest that, thanks to their high sampling frequency, long‐term monitoring data sets can enable detection of directional long‐term changes in parasite systems from background seasonal or interannual fluctuations. However, long‐term biodiversity changes related to anthropogenic changes may be captured best at the scale of several decades to a century, whereas long‐term monitoring studies have so far mostly been used to investigate relatively short timeframes [generally a decade or two, but see Zhokhov & Pugacheva (2021) for an exception] as they are resource intensive. Thus, researchers have favoured long‐term monitoring over other approaches when addressing questions requiring high temporal resolution but not a long study duration (e.g. to investigate parasite population dynamics or changes after a punctual ecosystem disturbance). However, our review indicates that understanding and effectively predicting how anthropogenic changes influence parasite infection levels and diversity requires allocating sufficient resources to sustain long‐term monitoring efforts of parasite systems extending over longer time periods and to foster the development of new large‐scale programmes. This is further supported by calls from other fields highlighting the overall need for long‐term ecological monitoring to improve our understanding of ecological processes and to allow informed decisions in terms of ecosystem management (Giron‐Nava *et al*., 2017; Lindenmayer, Lavery & Scheele, 2022; Hollister, 2024). As these large‐scale efforts require substantial resource investment, an important challenge of parasite ecology is to find ways to make long‐term parasite monitoring more efficient and cost‐effective. Integrating parasite monitoring into existing ecological monitoring programmes should enable minimising additional investment while maximising the amount of ecological information and studying ecosystems in a more holistic way. Additionally, the effort required to detect and identify parasites may also be reduced by designing and adopting scalable biomonitoring tools based on molecular methods such as meta‐barcoding or environmental DNA (eDNA) approaches (e.g. Thomas *et al*., 2022; Hammoud *et al*., 2022; Cabodevilla *et al*., 2023; Hupało *et al*., 2025). A variety of molecular assays recently have been developed in that direction, but most of them specifically target a restricted set of parasite species, typically those of economic or medical relevance (e.g. Kamel *et al*., 2021). Furthermore, these tools do not (yet) enable monitoring of parasite infection levels in the studied systems, and their cost sometimes remains prohibitive for application to biodiversity monitoring programmes. Thus, we advocate here for the development of holistic approaches to characterise parasite faunas from environmental or host samples in a cost‐efficient way.

The second most common type of studies, snapshot resampling, enables characterising changes in parasite populations or assemblages in the absence of long‐term monitoring programmes by leveraging existing historical data in a highly resource‐effective way. Performing contemporary sampling during the same season as historical sampling and employing the same methods is key to ensure data comparability (Quinn *et al*., 2021), but even so, this approach is constrained by its poor temporal resolution. Indeed, only two time points are often compared (i.e. historical *versus* contemporary), with sometimes little or no sampling replication at either or both of these ‘snapshots’ (i.e. only one host collection event at each time point). This low sampling frequency may lead to any long‐term changes identified reflecting seasonal or interannual fluctuations rather than actual directional long‐term changes and distinguishing between these scenarios may be much harder than when using long‐term monitoring data sets. Ensuring that enough time points or locations are covered at each ‘snapshot’ would mitigate this issue from a statistical perspective. However, it still leaves open the possibility that temporal differences observed may reflect extremes in stochastic or cyclic fluctuations rather than long‐term directional trends of parasite populations. Therefore, researchers studying parasite changes using a snapshot resampling methodology should validate their methodology and evaluate the robustness of their conclusions. This could be achieved by simulating snapshot resampling data sets from long‐term monitoring data using iterative resampling, before assessing the long‐term trends between these points and comparing the patterns observed with those emerging from the analysis of the entire long‐term monitoring data set. Given the high resource efficiency of snapshot resampling, we highly recommend researchers to adopt this approach whenever historical parasitological data are available.

A substantial number (>40%) of studies relying on natural history collections were aimed at tracking the spread of the invasive pathogenic amphibian chytrid fungus *Batrachochytrium dendrobatidis* (e.g. Talley *et al*., 2015; Fong *et al*., 2015) – which is causing conservation concerns in amphibian populations (Fisher & Garner, 2020). However, recent work also indicates that natural history collections offer unique opportunities to investigate changes in parasite infection levels and diversity on longer timescales than any other approach (Harmon *et al*., 2019; Preisser *et al*., 2022; Wood *et al*., 2023a; Wood & Vanhove, 2023). Characterising changes in parasite infections from preserved host specimens may therefore open the possibility to study how anthropogenic activities have affected host–parasite systems at the scale of a century, even in the absence of historical studies (Fiorenza *et al*., 2020; Wood *et al*., 2023b). As such analyses have mostly been performed in North America, we suspect that there is an untapped potential for natural history collection‐based research in other continents and especially Europe given the longstanding tradition of preserving natural history specimens. Our results, however, suggest that such studies may face important methodological limitations. Firstly, the resolution of parasite identification is poorer than in the other approaches (Figs 5 and S11). This is likely because (*i*) insufficient resources are allocated to train researchers to identify specimens morphologically, (*ii*) traditional fixatives degrade DNA and therefore hinder molecular identification methods, and (*iii*) key characteristics for morphological identification are often lost or altered after preservation, which further limits the resolution of specimen identification without molecular approaches. Secondly, studies based on natural history collections generally rely on lower sample sizes, most likely due to low availability of specimens in collections. Thus, patterns arising from these studies should ideally be corroborated with evidence obtained from other approaches (e.g. Howard *et al*., 2019). In addition, utilising natural history collections in long‐term parasite assessments can often result in the partial or complete destruction of the preserved host specimens, thereby compromising the information they hold for future use (Wood *et al*., 2023a; Wood & Vanhove, 2023). Furthermore, it remains unclear whether studies based on natural history collections can help elucidate long‐term trends in all parasite–host systems. Currently efforts have been limited to amphibians and fish, likely because these organisms tend to be preserved in large numbers and as whole specimens in liquid preservatives, formalin or ethanol.

Based on this, we identify two prerequisites to use natural history collections for long‐term parasite studies: (*i*) the host preservation method should allow parasites to be preserved in a state that permits identification to a meaningful taxonomic level; and (*ii*) suitable host information and metadata should be available for analysis (see Wood *et al*., 2023a). Due to the potentially destructive nature of parasite sampling from hosts preserved in natural history collections, what constitutes a reasonable sample size should ideally be assessed beforehand using power analyses. While we can expect these two criteria to be met often for amphibian, fish and reptile hosts, mammals and birds are usually taxidermized and are rarely preserved with their internal organs or in sufficient numbers (Wood *et al*., 2023a). Therefore, we propose that further research should aim at easing these bottlenecks to ensure that natural history collections provide high‐quality long‐term parasite data. To achieve this, improvement of identification techniques of preserved parasites, while limiting the destructive impact on their hosts should be a priority. Recent work points towards the future possibility of using molecular assays to analyse formalin‐preserved specimens (Vivien, Ferrari & Pawlowski, 2016; Hou *et al*., 2021; Brino *et al*., 2023), which may improve the resolution of parasite identification, but would come at the cost of destroying (parts of) preserved host and parasite specimens for dissection and DNA extraction.

Finally, collating parasite data from multiple published sources – whether collected with a long‐term aim or not – offers the possibility to detect large‐scale trends in parasite distribution, infection levels, or diversity. Our results show that such literature‐based studies allow exploring trends over long time spans, typically with large sample sizes. This approach presents the advantages of not requiring additional field sampling, increasing resource efficiency. Literature‐based reviews and syntheses are, however, limited by the amount and quality of data accessible, and they may combine data collected using different diagnostic methods. Thus, ensuring that parasitological data produced through monitoring, resampling or natural history collection examination becomes available for use by the scientific community is paramount, both to provide baseline data for resampling studies and to reveal patterns across individual long‐term trends at the synthesis level. Therefore, we strongly recommend that authors adhere to the principles of findability, accessibility, interoperability, and reusability (FAIR) when making their data available (Wilkinson *et al*., 2016b). Given the surprisingly large number of long‐term parasite studies uncovered by this review, there may be untapped potential for quantitative syntheses of temporal changes in parasite distribution patterns, abundance and diversity similar to those conducted for free‐living organisms (e.g. Dornelas *et al*., 2014; Rosenberg *et al*., 2019).

### (3) Broad trends in parasite populations and assemblages

Our semi‐quantitative assessment of temporal trends did not reveal any overarching directional trends in long‐term changes in parasite infection levels or diversity. The number of studies reporting observable directional changes in parasite populations or assemblages was roughly equal to those reporting no significant change, and where changes were noted, increases and decreases in parasite infection levels or diversity were approximately equally frequent. Depending on the parameter assessed, this situation differs from observations in free‐living groups. For example, Lees *et al*. (2022) showed that many biodiversity indicators point towards a loss of bird diversity globally. By contrast, while other authors report no loss of alpha diversity in other taxonomic groups, they note instead a trend of high compositional turnover and/or homogenisation resulting in reduced beta diversity (Dornelas *et al*., 2014; Finderup Nielsen *et al*., 2019; Rishworth *et al*., 2020). The apparent lack of overarching trends in parasites may reflect idiosyncratic or no responses to anthropogenic environmental changes. Alternatively, the data compiled in our review might not be enough to unveil global patterns of temporal changes. Finally, our summary of the findings of these studies, which amounts to vote counting, is admittedly not the most appropriate way of identifying such overarching trends. Therefore, in addition to advocating for increased efforts aimed at long‐term data generation, we recommend working towards synthesising evidence on long‐term changes in parasite systems using meta‐analytical methods. Although meta‐analyses of large‐scale, long‐term trends in parasite populations have been performed in the past (Fiorenza *et al*., 2020; Mastick, Fiorenza & Wood, 2024a), such studies are extremely rare and generally focus on a handful of species or genera – therewith limiting comparability across taxonomic groups. We believe that characterising large‐scale changes in parasite infection levels across multiple parasite groups would provide crucial insights into global trends in parasite populations – or lack thereof – and into how different parasite groups are coping with anthropogenic changes. Such syntheses may be achievable based on the substantial number of long‐term studies focusing on parasite infection levels and because diversity‐focused studies often generate infection levels data. At a glance, we estimate that our data set includes trajectories of >2,000 parasite populations. This number could be sufficient for a quantitative synthesis, although it is considerably smaller than those corresponding to well‐curated databases for free‐living populations [e.g. The Living Planet Index database (LPI, 2023) contains data on ~42,000 population trajectories of vertebrate species] to assess potential temporal changes in functional traits in parasite assemblages, provided that trait data are available for the species at hand (Llopis‐Belenguer *et al*., 2019; Poulin, 2021). For parasite diversity however, the limited availability of data sets may limit the applicability of meta‐analyses. Additionally, the temporal resolution of reported changes in parasite diversity was overall lower than that for parasite infection levels as the most common way of approaching changes in diversity was through snapshot resampling, whereas changes in infection levels were more often characterised through long‐term monitoring. Therefore, attempts to analyse trends in parasite diversity through meta‐analyses may be hampered by the paucity of studies and of temporal sampling points per studied system.

Finally, we observed that the reported links between anthropogenic drivers and changes were rarely assessed using inferential analyses but rather were often suggested *a posteriori*. Similarly, the causes of frequent apparent stability in the parasite system studied were rarely addressed. The observed rarity of inferential analyses applied to testing links between temporal changes in parasite systems and anthropogenic disturbance is most likely related to the difficulty – and often impossibility – of disentangling the various interacting environmental and ecological factors affecting parasites over extended periods of time based on observational data only. Furthermore, the availability of accurate historical information on anthropogenic changes can be limiting in itself. While we fully acknowledge these limitations, we encourage authors to continue to work towards characterising temporal changes in parasite systems and to test links with anthropogenic drivers using correlational analyses. In combination with insights gained from laboratory‐, mesocosm‐, or field‐based experimental approaches, which are incapable of replicating the intertwined impacts of changing ecologies over decades but can inform on the mechanisms at play, these findings will be key to advancing our understanding of the observed changes in parasite systems.

### (4) Observed gaps and biases

Nearly 75% of the long‐term studies analysed were conducted in Europe or North America, despite these continents covering only about 24% of Earth's land surface. Admittedly, some non‐English or grey literature originating from less well‐represented regions may have eluded our systematic search as they may not be indexed or findable using English search terms. However, as similar patterns are observable in the availability of diversity data from free‐living organisms, we strongly believe that the observed high representation of studies focused on European and North American ecosystems reflects real biases in research efforts (Magurran *et al*., 2010; Trimble & Aarde, 2012). Addressing these geographical biases is not only key for capturing how parasite systems are changing at global scale, but also for identifying region‐specific patterns. Strikingly, we observe that long‐term wildlife parasite data are scarce in areas most affected by climate change, such as the Arctic (Rantanen *et al*., 2022) or in biodiversity hotspots, such as the Neotropics, subtropical Africa and South‐East Asia (Jenkins, Pimm & Joppa, 2013). As the diversity of parasite faunas correlates with the diversity of potential hosts (Hechinger & Lafferty, 2005; Thieltges *et al*., 2011; Kamiya *et al*., 2014; Johnson *et al*., 2016), biodiversity hotspot areas are also expected to contain highly diverse parasite assemblages. Assessing the past and future of the high, although largely uncharted, diversity of parasites in these regions thus remains impossible due to the lack of long‐term and in‐depth data (due to both high diversity and low study effort). As very few long‐term monitoring studies have taken place in tropical areas, we suggest that snapshot resampling and natural history collection‐based studies provide an opportunity to look back in time and investigate how parasite diversity has changed over the last century in these regions. With a few exceptions (e.g. Chapman *et al*., 2012), historical data sets on the diversity of parasite assemblages in tropical regions are rare. However, several museum and other natural history institutions hold preserved host specimens originating from tropical regions that may be used to characterise past parasite assemblages (e.g. Jorissen *et al*., 2020). Despite the limitations of this approach (see Section IV.2 and Table 1), natural history collections may represent the only source of historical data in systems that have not been studied by parasitologists in the past. We therefore encourage researchers to keep providing new specimens to museums and to use available preserved specimens to assess long‐term trends in these systems. However, we acknowledge that the poor taxonomic resolution currently characterising natural history collection‐based studies may limit the depth and interpretability of the patterns highlighted, especially in regions likely to harbour high uncharted parasite taxonomic diversity. Importantly, as many specimens may have been collected in the context of colonialism, they might currently be stored in institutions located in the Global North. We strongly believe that researchers from these institutions should only work on these samples in the context of collaborations with scientists from the country of origin of the samples. Indeed, knowledge from local scientists and stakeholders may be crucial to gather relevant data and successfully interpret long‐term changes in parasite systems in the context of regional historical changes (Yanou *et al*., 2023; Pardoe *et al*., 2024). Importantly, sharing the benefits of these research outputs and including regional expertise in long‐term parasitological research will be key to engaging in more inclusive and decolonised research pathways.

Our results show that ~72% of long‐term studies focused on fish, mammal, or bird hosts. Studies on terrestrial ecosystems were mainly based on mammalian and bird hosts, focusing on helminths, arthropods (mostly ticks) or protists (mostly in birds and reptiles). Contrastingly, studies on aquatic ecosystems overwhelmingly focused on fish and molluscs and their helminth or arthropod parasites. Data on other metazoan host groups (e.g. insects and crustaceans), which represent the largest share of animal diversity, are scarce, limiting comparisons of patterns across groups. Furthermore, a sizeable proportion of parasite species use complex life cycles to reproduce, infecting hosts from distant taxonomic and functional groups at distinct stages of their life. Our knowledge of most parasite life cycles is fragmentary, but integrated molecular monitoring approaches may complement traditional experimental approaches to help researchers fill this gap (Blasco‐Costa & Poulin, 2017; Bennett, Presswell & Poulin, 2023). Despite the considerable effort required to analyse parasite dynamics in all hosts involved in their life cycles, doing so provides invaluable insights into the mechanisms responsible for observed population and diversity changes. Indeed, observed temporal changes in parasite systems can originate from processes acting on free‐living larvae and/or on one or multiple hosts (Pietrock & Marcogliese, 2003; Sures *et al*., 2023). Gathering long‐term parasite data from multiple hosts in the same ecosystem, as well as on free‐living stages should therefore provide better insights into the driving ecological processes of change. As parasite transmission between hosts involved in the parasite's life cycle often occurs through trophic interactions, reproductive adult parasites are generally found in hosts occupying high trophic levels, whereas larval stages are more common in hosts of low trophic levels (Benesh *et al*., 2021). We observed that long‐term studies on parasites with complex life cycles often focused on infections in one host species, or a few species of closely related hosts, meaning that long‐term research has rarely evaluated the trajectory of parasites in a holistic manner, by considering temporal changes in different life stages simultaneously (for a rare example see Kennedy & Rumpus, 1977). Instead, the literature is biased towards high‐trophic‐level hosts (fish, birds, or mammals), with low‐trophic‐level hosts (e.g. insects, crustaceans) poorly represented. Consequently, with the notable exception of larval trematodes within mollusc hosts which have been intensively studied, our understanding of long‐term dynamics of parasites is poorer for juveniles and adults.

Finally, obtaining accurate taxonomic information relying on harmonised classification systems is essential to any kind of biodiversity assessment, parasites being no exception. Parasite taxonomy as a discipline is currently at risk of losing invaluable expertise and traction due to the gradual loss and lack of replacement of active experts (Brooks & Hoberg, 2001; Poulin & Presswell, 2022). Our results suggest that molecular approaches may to some extent contribute to palliate this problem by ensuring high precision of parasite identification in diversity assessments (Scholz, 2024). Additionally, molecular approaches may also help to link parasites across life cycle stages, representing an efficient way to inform on parasite life cycles (Blasco‐Costa & Poulin, 2017). However, morphological data are required to describe new species fully (which may encompass the majority of parasites globally, and particularly in the tropics), and molecular methods are far from free of biases and inaccuracies (Scholz, 2024). For example, DNA barcoding is sensitive to identification errors in molecular databases and to the absence of closely matching sequences – an issue particularly common in some helminth groups (Schols *et al*., 2020). Therefore, to ensure high taxonomic resolution, we strongly recommend studies of temporal changes in parasite diversity to adopt integrative taxonomic approaches, where both morphological and molecular clues are simultaneously considered when possible (Dayrat, 2005; Blasco‐Costa & Poulin, 2017). To that aim, the maintenance of high‐quality training in parasite taxonomy is strongly encouraged, especially because morphological identification often remains the only option for specimens in natural history collections.

## V. CONCLUSIONS

(1) We identified four approaches deployed to gather long‐term data on the diversity or infection levels of wildlife animal parasites: (*i*) long‐term monitoring; (*ii*) snapshot resampling; (*iii*) literature‐based research; and (*iv*) natural history collection‐based studies. We observed significant differences in the temporal scope, geographical scale of sampling, sample sizes and taxonomic resolution of parasite identification among these approaches.
(2) Long‐term parasite research has primarily focused on European and North American ecosystems. This bias should be addressed by supporting research focused on understudied areas, many of which are parasite biodiversity hotspots or severely affected by climate change.
(3) Long‐term parasite research has mostly focused on vertebrate hosts. Including additional invertebrates in future assessments should enhance our understanding of long‐term changes in parasite transmission, especially for species with complex life cycles.
(4) General long‐term directional trends in parasite diversity remain elusive, even though such trends are expected as the ecosystems in which these parasites and their hosts coexist are undergoing significant changes. Although most ecosystems worldwide are being impacted by human alterations, the long‐term consequences of anthropogenic disturbances on parasitic organisms remain poorly understood, thereby limiting our capacity to predict the potential feedback of changes in parasite systems on ecosystems themselves. Disentangling these effects and characterising trajectories of parasite diversity and infection levels requires parasite research to be brought up to speed with its free‐living counterparts.
(5) Lack of historical information and poorly resolved knowledge on the ecology, life cycles or taxonomy of most parasite taxa currently hampers our understanding of long‐term patterns and dynamics of parasite systems, and their interactions with anthropogenic disturbances. To fill these data gaps, we will need to intensify our efforts to gather diversity and infection level data. To that end, all approaches of field data collection discussed herein may find value. Ultimately, integrating long‐term parasite data into large‐scale quantitative syntheses will provide the most robust evidence of human‐driven changes in parasite populations and assemblages – or lack thereof.

## Supporting information


**Table S1.** Long‐term wildlife animal parasite bibliographic data set compiled through our study.

**Table S2.** Structure and results of the statistical tests performed to investigate differences among research approaches.
**Table S3.** Proportion of studies that provide data on the different parasite groups, per host group.
**Fig. S1.** PRISMA chart of the systematic bibliographic search performed.
**Fig. S2.** The timespan and scale of geographical scope of studies according to the research approach implemented.
**Fig. S3.** Map of the geographical location of studies in Europe and North America.
**Fig. S4.** Plot matrix using the Pearson residuals of the Chi‐squared test showing that the distribution of studies among continents differs according to the research approach used.
**Fig. S5.** Representation of the type of environment studied according to the research approach implemented.
**Fig. S6.** Number of host and parasite taxa analysed in 233 studies, grouped by research approach.
**Fig. S7.** Relationship between the number of parasite taxa analysed and the number of different host organs inspected across 216 studies, grouped by research approach.
**Fig. S8.** Distribution of host types among type of environment studied.
**Fig. S9.** Distribution of parasite types among type of environment studied.
**Fig. S10.** Taxonomic resolution of parasite identification depending on the broad identification method.
**Fig. S11.** Summary of taxonomic resolution of parasite identification depending on both research approach and identification method.

## Data Availability

The data that support the findings of this study are openly available in Zenodo at https://doi.org/10.5281/zenodo.17856798.
