# Supplementary material for: Long‐term trends in parasite diversity and infection levels: approaches and patterns
Source: Biol Rev Camb Philos Soc. 2025 Dec 19;101(3):1120–42. doi: 10.1002/brv.70119 (PMC13149780; doi:10.1002/brv.70119)
Supplement: Supplementary file 2 — Table S2. Structure and results of the statistical tests performed to investigate differences among research approaches. Table S3. Proportion of studies that provide data on the different parasite groups, per host group. Fig. S1. PRISMA chart of the systematic bibliographic search performed. Fig. S2. The timespan and scale of geographical scope of studies according to the research approach implemented. Fig. S3. Map of the geographical location of studies in Europe and North America. Fig. S4. Plot matrix using the Pearson residuals of the Chi‐squared test showing that the distribution of studies among continents differs according to the research approach used. Fig. S5. Representation of the type of environment studied according to the research approach implemented. Fig. S6. Number of host and parasite taxa analysed in 233 studies, grouped by research approach. Fig. S7. Relationship between the number of parasite taxa analysed and the number of different host organs inspected across 216 studies, grouped by research approach. Fig. S8. Distribution of host types among type of environment studied. Fig. S9. Distribution of parasite types among type of environment studied. Fig. S10. Taxonomic resolution of parasite identification depending on the broad identification method. Fig. S11. Summary of taxonomic resolution of parasite identification depending on both research approach and identification method. [file BRV-101-1120-s001.docx]

**SUPPORTING INFORMATION**

**Table S1.** (see separate document: TableS1.csv) Long-term wildlife animal parasite bibliographic data set compiled through our study. Also available in Zenodo at https://doi.org/10.5281/zenodo.17856798, along with the R script used to perform statistical analyses and create figures. Field description:

- study: bibliographic reference of the long-term parasite study.
- language: language of the publication.
- publication_year: year of publication of the article.
- decade: decade of publication of the article.
- temporal_span: span, in years, covered by the study (earliest and latest years included).
- start: year of the earliest relevant sampling event in the study.
- end: year of the latest relevant sampling event in the study.
- lat: latitude of the approximate centroid of sampling of the study (WGS84, decimal degrees).
- long: longitude of the approximate centroid of sampling of the study (WGS84, decimal degrees).
- continent: continent where the study took place.
- organs_n: number of different host organs inspected for parasite infections.
- num_host_taxa: number of different host taxa included in the study.
- host_taxon_gen_bis: taxonomic group to which the host included in the study belong.
- n_hosts: total host sample size (in number of individuals screened for parasite infections).
- num_parasite_taxa: number of different parasite taxa included in the study.
- helminth: if yes, helminths were among the parasites included in the study.
- arthropod: if yes, arthropods were among the parasites included in the study.
- myxozoa: if yes, Myxozoa were among the parasites included in the study.
- hirudinea: if yes, leeches were among the parasites included in the study.
- protozoa: if yes, protozoa were among the parasites included in the study.
- mycete: if yes, mycetes were among the parasites included in the study.
- environment: broad type of environment in which the study took place.
- geo_scale: broad geographical scale of the study.
- method_study: methodological approach deployed to investigate parasites in the long term.
- secondary_method: any complementary methodological approach employed to investigate parasites in the long term.
- method_parasite_ID: method used to identify parasites.
- aim_general: broad aim of the study.
- ID_sp: proportion of parasite taxa included in the study that were identified to species level or with more precision (strain, subspecies, etc.).
- ID_gen: proportion of parasite taxa included in the study that were identified to genus level.
- ID_fam_higher: proportion of parasite taxa included in the study that were identified at lower resolution than genus level.
- prop_larval: proportion of parasite taxa included in the study that were at the larval stage.
- hypo_clim: was climate change hypothesized to drive long-term changes in parasite infection levels?
- test_clim: were links between changes in parasite infection levels and climate change tested?
- hypo_eutro: was eutrophication hypothesised to drive long-term changes in parasite infection levels?
- test_eutro: were links between changes in parasite infection levels and eutrophication tested?
- hypo_intro: was the introduction of alien species hypothesised to drive long-term changes in parasite infection levels?
- test_intro: were links between changes in parasite infection levels and alien species introduction tested?
- hypo_habitat: were habitat changes hypothesised to drive long-term changes in parasite infection levels?
- test_habitat: were links between changes in parasite infection levels and habitat changes tested?
- hypo_pollutant: was pollution hypothesised to drive long-term changes in parasite infection levels?
- test_pollutant: were links between changes in parasite infection levels and pollution tested?
- hypo_protect: were measures of environmental protection hypothesised to drive long-term changes in parasite infection levels?
- test_protect: were links between changes in parasite infection levels and measures of environmental protection tested?
- change_alpha: what changes in the alpha diversity of parasite did the authors report (if any)?
- turnover_comp: did the authors report changes in the composition of parasite assemblages?
- abundance_change: what changes in infection levels of parasite did the authors report (if any)?

**Table S2.** Structure and results of the statistical tests performed to investigate differences among research approaches. d.f. = degrees of freedom.

|  | **Type** | **Model** | ***N*** | **Term** | ***P value*** |
| --- | --- | --- | --- | --- | --- |
| T1 | Pairwise Wilcoxon test | Study span ~ research approach | 244 | Long-term monitoring *vs* Snapshot resampling | **< 0.001** |
|  |  |  |  | Long-term monitoring *vs* Literature data | **< 0.001** |
|  |  |  |  | Long-term monitoring *vs* Natural history collections | **< 0.001** |
|  |  |  |  | Snapshot resampling *vs* Literature data | **< 0.001** |
|  |  |  |  | Snapshot resampling *vs* Natural history collections | **< 0.001** |
|  |  |  |  | Literature data *vs* Natural history collections | **< 0.010** |
| T2 | Chi-squared test | Number of studies in continent ~ research approach | 244 | Research approach | **< 0.001** (d.f. = 21,  *X^2^* = 100.7) |
| T3 | Chi-squared test | Number of studies in geographical scale category ~ research approach | 244 | Research approach | **< 0.001** (d.f. = 6,  *X^2^* = 47.3) |
| T4 | Chi-squared test | Number of studies in environment category ~ research approach | 244 | Research approach | **< 0.001** (d.f. = 12, *X^2^* = 70.0) |
| T5 | Chi-squared test | Number of studies in host taxon category ~ research approach | 244 | Research approach | **< 0.001** (d.f. = 24, *X^2^* = 116.4) |
| T6 | Chi-squared test | Number of identifications in taxonomic resolution category ~ research approach | 2316 | Research approach | **< 0.001** (d.f. = 6,  *X^2^* = 137.6) |
| T7 | Chi-squared test | Number of studies in research aim category ~ research approach | 244 | Research approach | **< 0.001** (d.f. = 12, *X^2^* = 73.5) |
| T8 | Chi-squared test | Number of identifications in taxonomic resolution category ~ identification method | 2316 | Identification method | **< 0.001** (d.f. = 4,  *X^2^* = 27.2) |
| T9 | Pairwise Wilcoxon test | Time adjusted host sample size ~ research approach | 214 | Long-term monitoring *vs* Snapshot resampling | **< 0.001** |
|  |  |  |  | Long-term monitoring *vs* Literature data | > 0.100 |
|  |  |  |  | Long-term monitoring *vs* Natural history collections | **< 0.001** |
|  |  |  |  | Snapshot resampling *vs* Literature data | **< 0.050** |
|  |  |  |  | Snapshot resampling *vs* Natural history collections | **< 0.001** |
|  |  |  |  | Literature data *vs* Natural history collections | **< 0.010** |

|  | Helminth (*N* = 171) | Arthropod (*N* = 58) | Protist  (*N* = 49) | Fungus (*N* = 17) | Myxozoan (*N* = 17) | Leech (*N* = 8) |
| --- | --- | --- | --- | --- | --- | --- |
| Fish (*N* = 93) | 55.3% | 21.3% | 8.7% | 0.7% | 9.3% | 4.7% |
| Mammal (*N* = 51) | 64.2% | 20.8% | 15.1% | 0.0% | 0.0% | 0.0% |
| Bird (*N* = 31) | 23.5% | 23.5% | 52.9% | 0.0% | 0.0% | 0.0% |
| Mollusc (*N* = 29) | 84.8% | 3.0% | 9.1% | 0.0% | 3.0% | 0.0% |
| Multiple (*N* = 15) | 59.1% | 13.6% | 9.1% | 9.1% | 4.5% | 4.5% |
| Amphibian (*N* = 13) | 7.7% | 0.0% | 0.0% | 84.6% | 7.7% | 0.0% |
| Crustacean (*N* = 4) | 28.6% | 28.6% | 14.3% | 28.6% | 0.0% | 0.0% |
| Insect (*N* = 4) | 50.0% | 0.0% | 25.0% | 25.0% | 0.0% | 0.0% |
| Reptile (*N* = 4) | 0.0% | 25.0% | 75.0% | 0.0% | 0.0% | 0.0% |

**Table S3.** Proportion of studies that provide data on the different parasite groups, per host group. Sample sizes represent the number of studies reporting data on the specific host or parasite group.

**
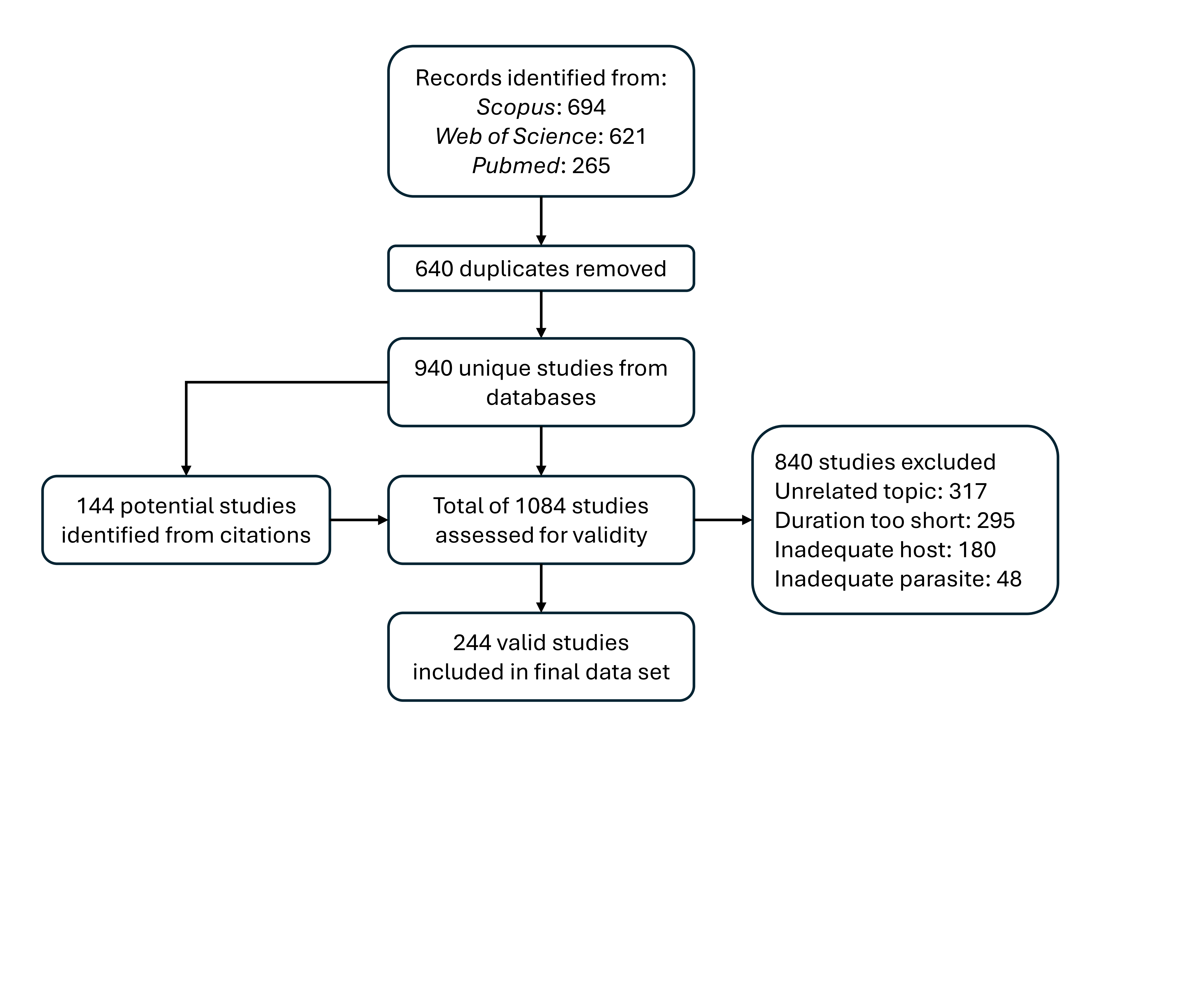
Fig. S1.** PRISMA chart of the systematic bibliographic search performed.

**
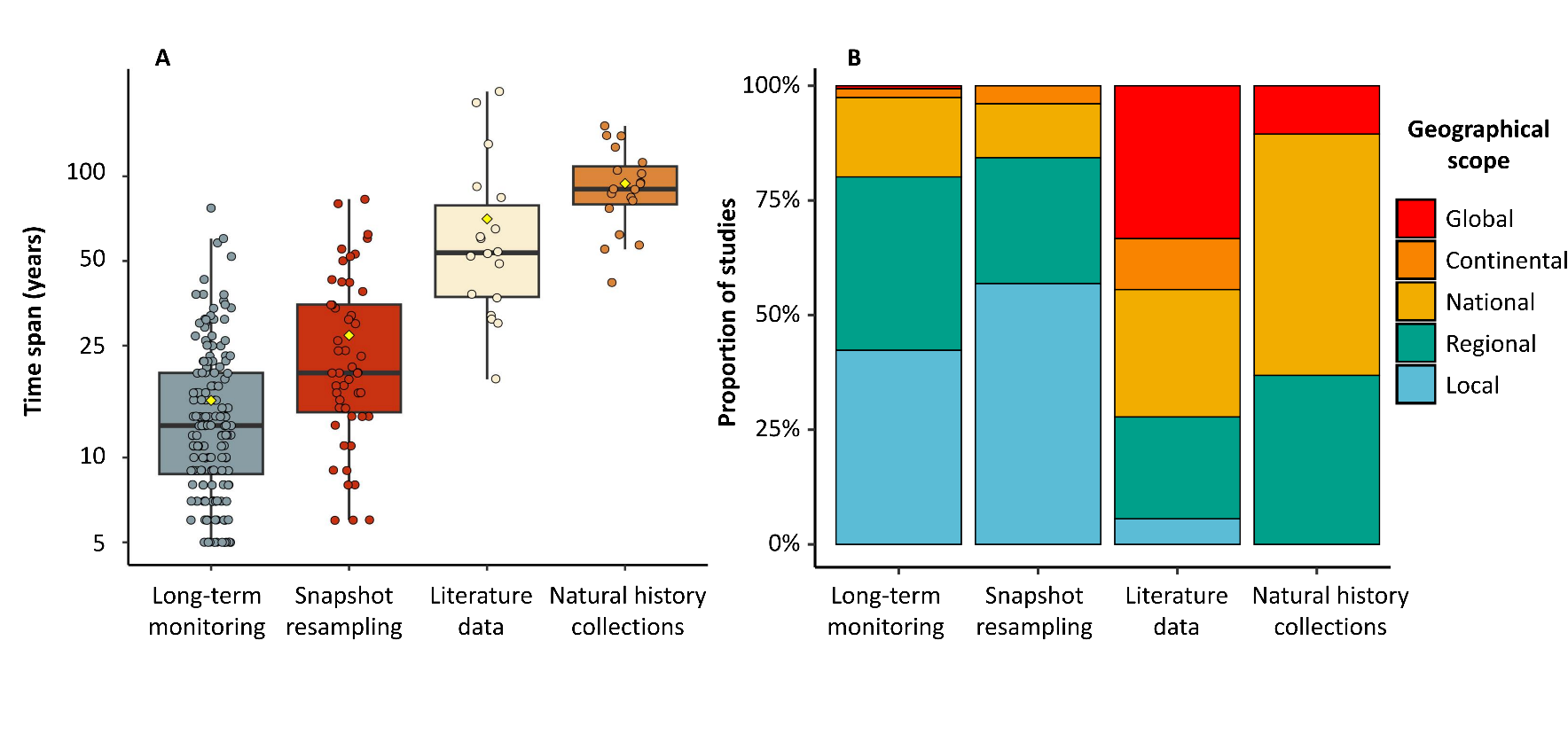
Fig. S2.** (A) Boxplot of the time span covered by studies according to the research approach implemented. Yellow diamonds show the mean time span of studies in each category, while thick lines and box limits indicate median and quartiles, respectively. The *y*-axis is log-transformed to facilitate readability. (B) Scale of the geographical scope of studies according to the research approach implemented.





**Fig. S3.** Map of the geographical location of long-term studies in (A) Europe and (B) North America. Geographic coordinates are the approximate centroid of the study area. Points are coloured by approach and slightly jittered to avoid excessive overlap.

**
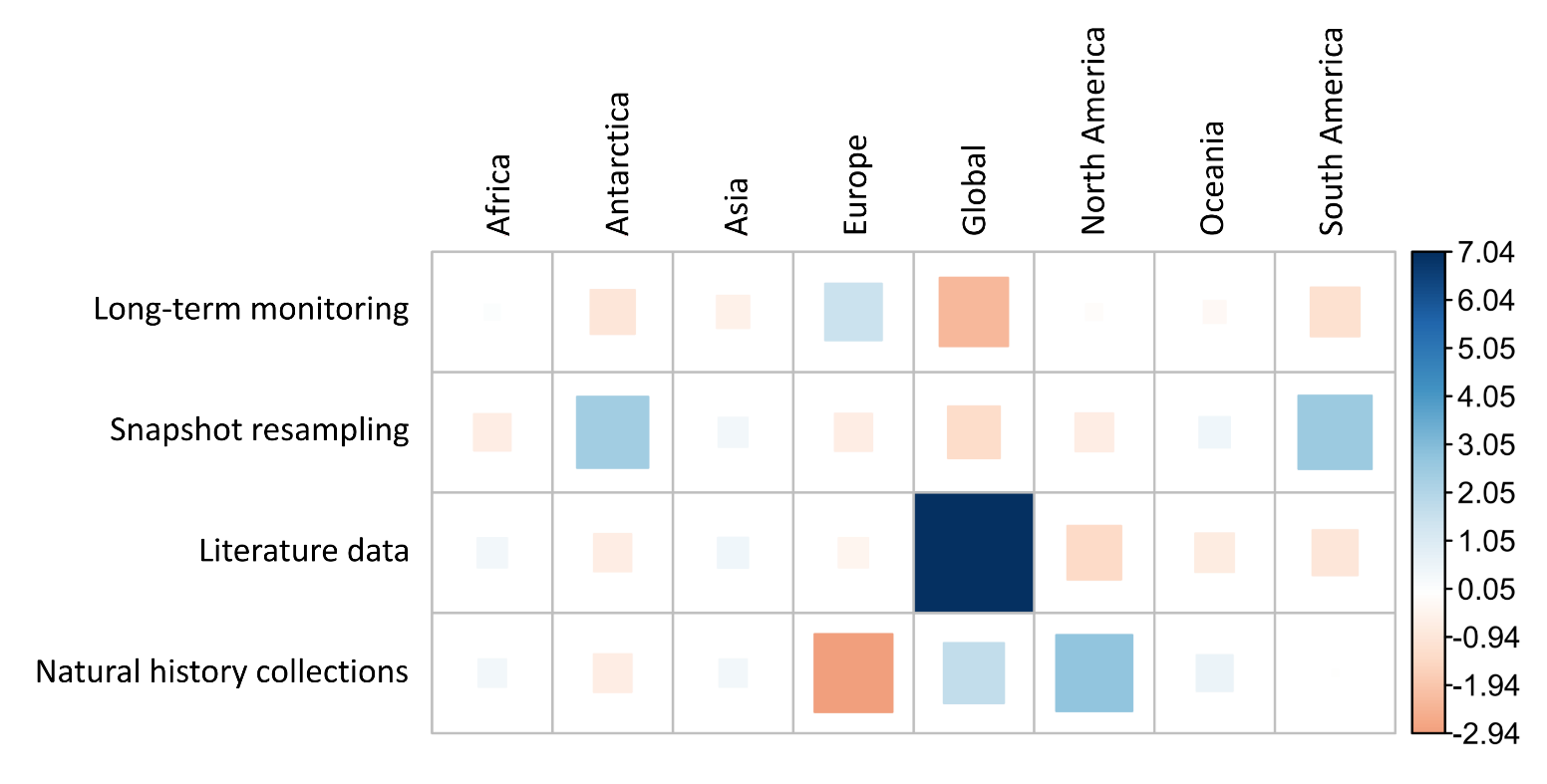
Fig. S4.** Plot matrix using the Pearson residuals of the Chi-squared test (Table S2, T2), showing that the distribution of studies among continents differs according to the research approach used.

**
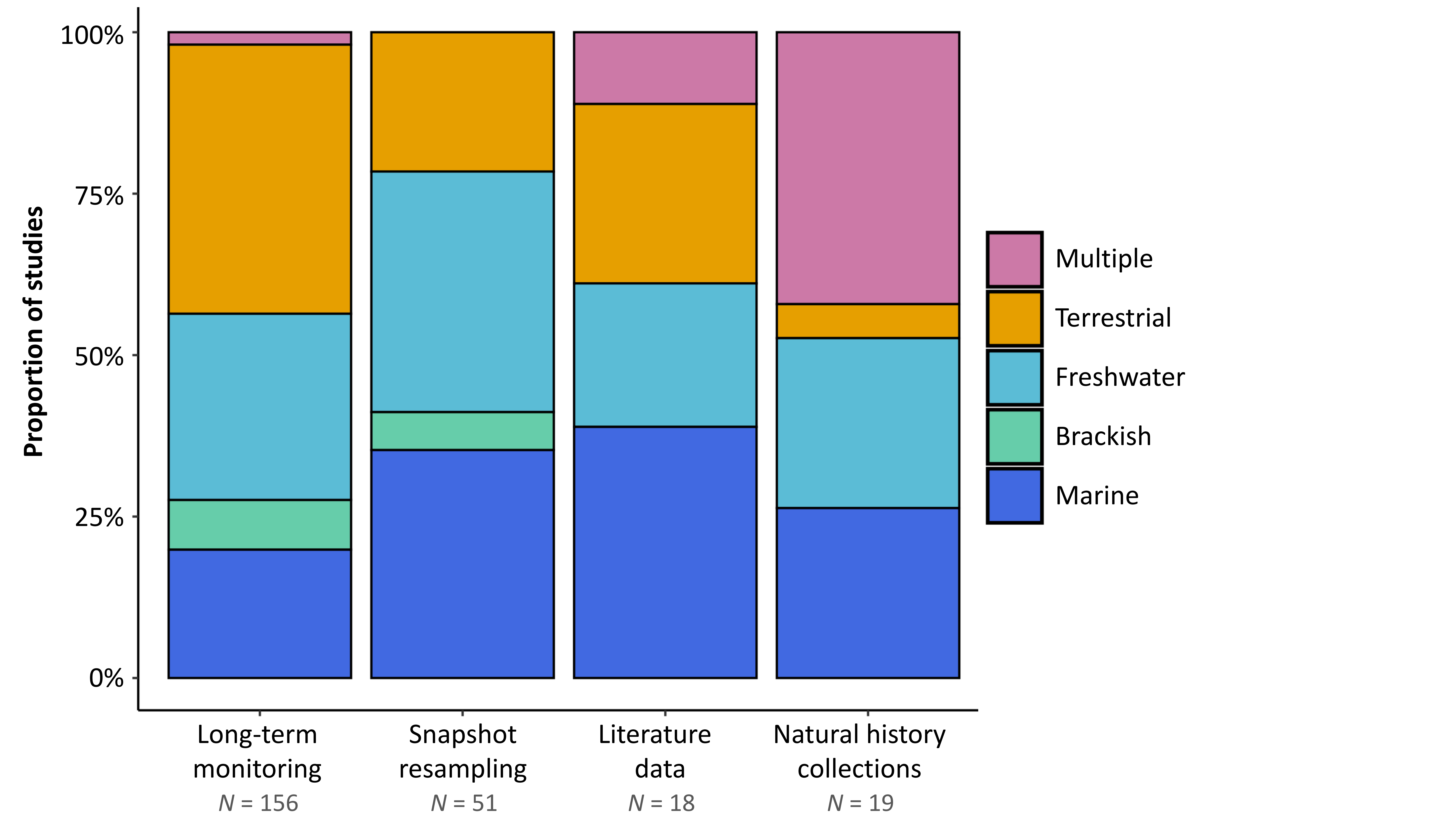
Fig. S5.** Representation of the type of environment studied according to the research approach implemented.

**
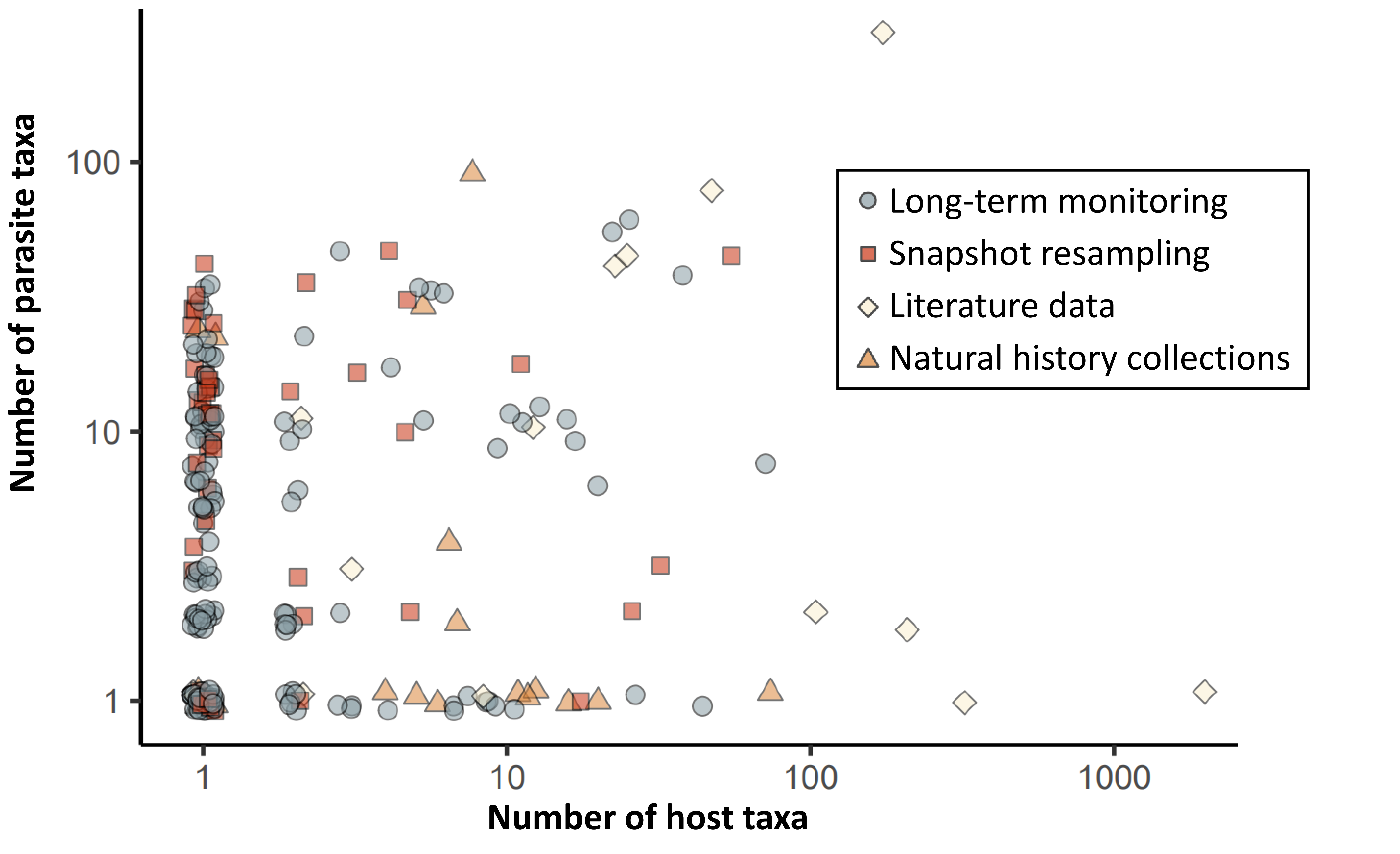
Fig. S6.** Number of host and parasite taxa analysed in 233 studies, grouped by research approach (11 studies did not provide information on the number of either host or parasite taxa studied).

**
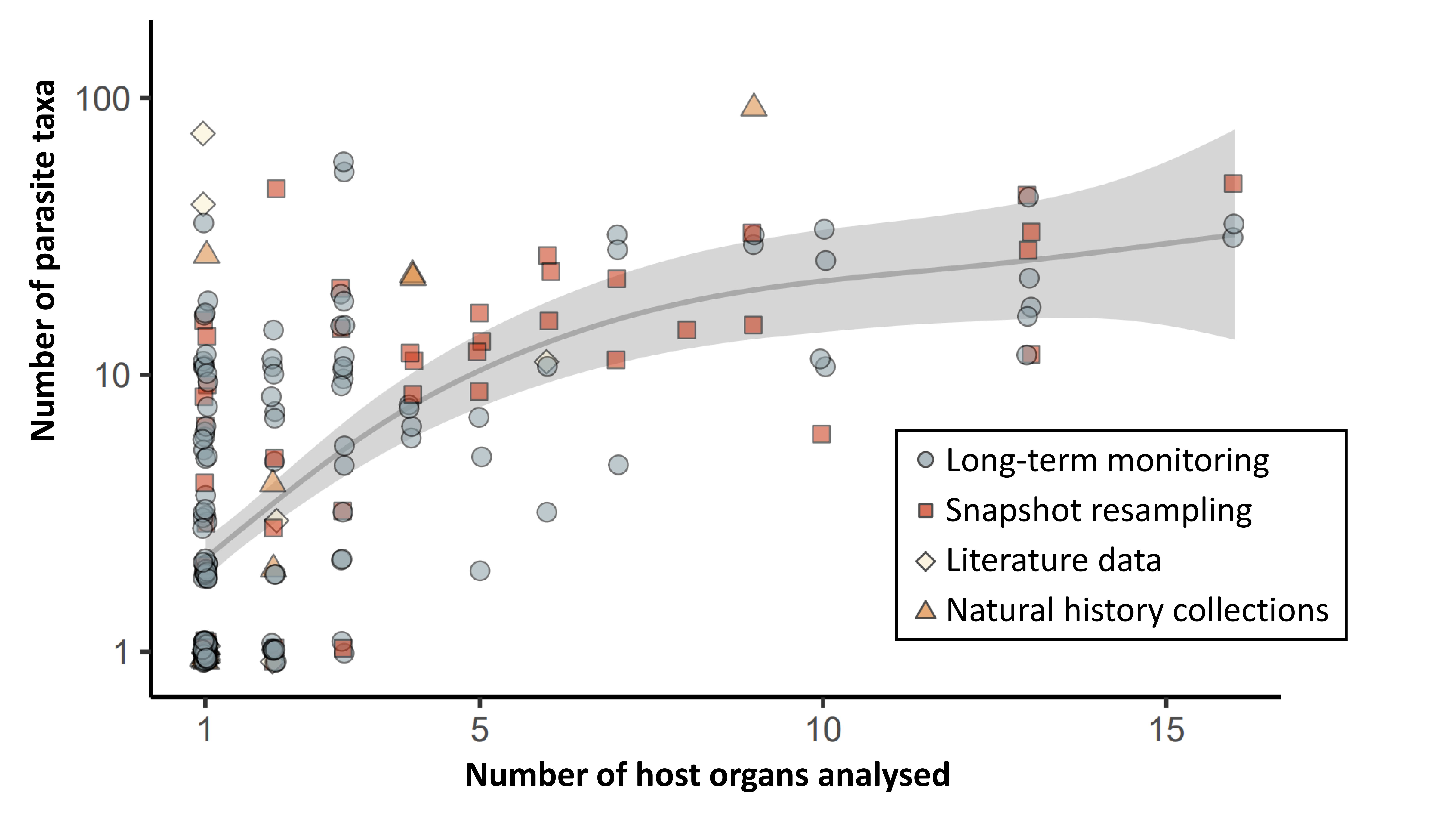
Fig. S7.** Relationship between the number of parasite taxa analysed and the number of different host organs inspected across 216 studies, grouped by research approach (28 studies did not provide information on either the number parasite taxa or which organs were screened for infections). A smooth curve based on generalised additive modelling is fitted to illustrate the trend.

**
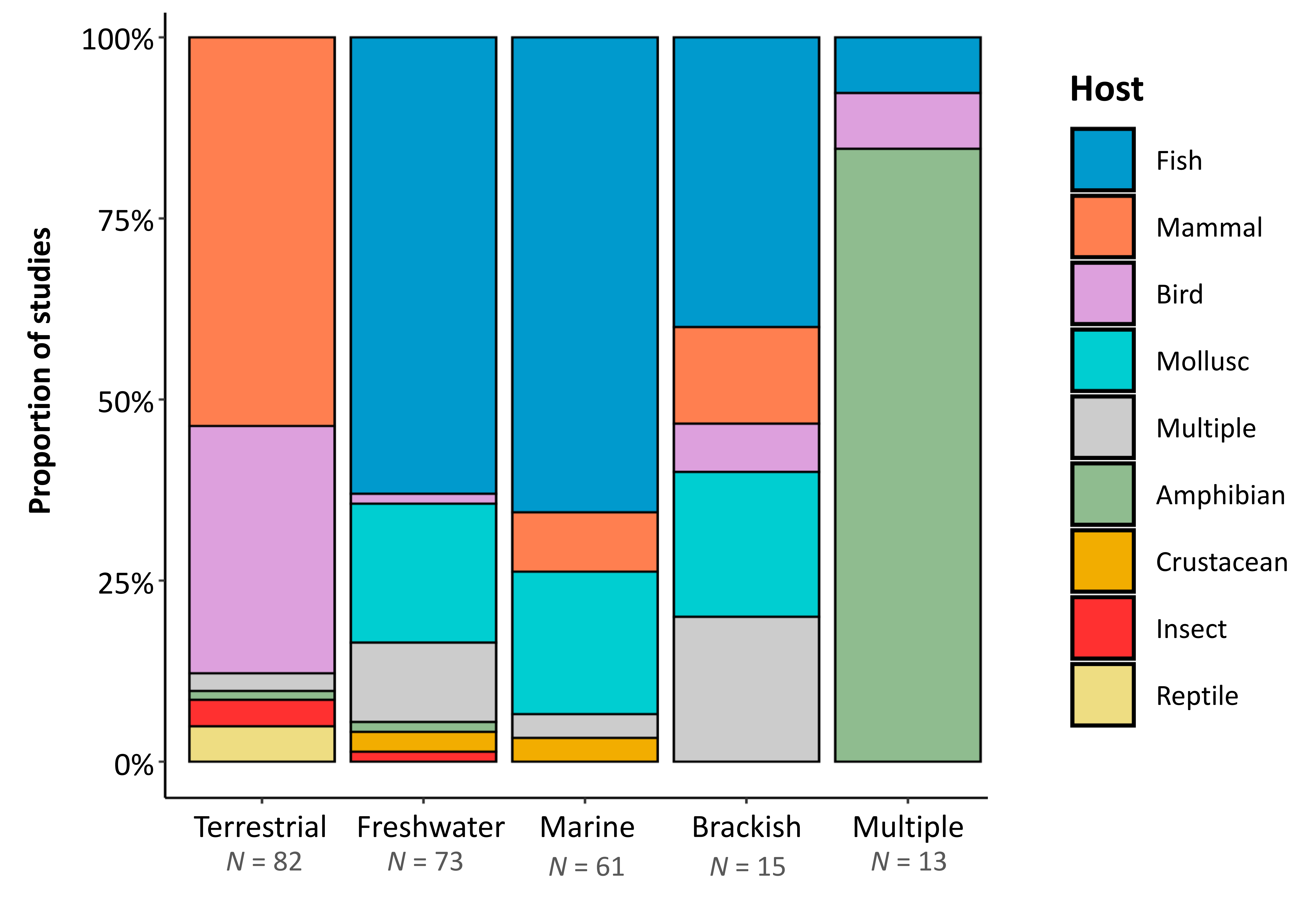
Fig. S8.** Distribution of host types among type of environment studied.

**
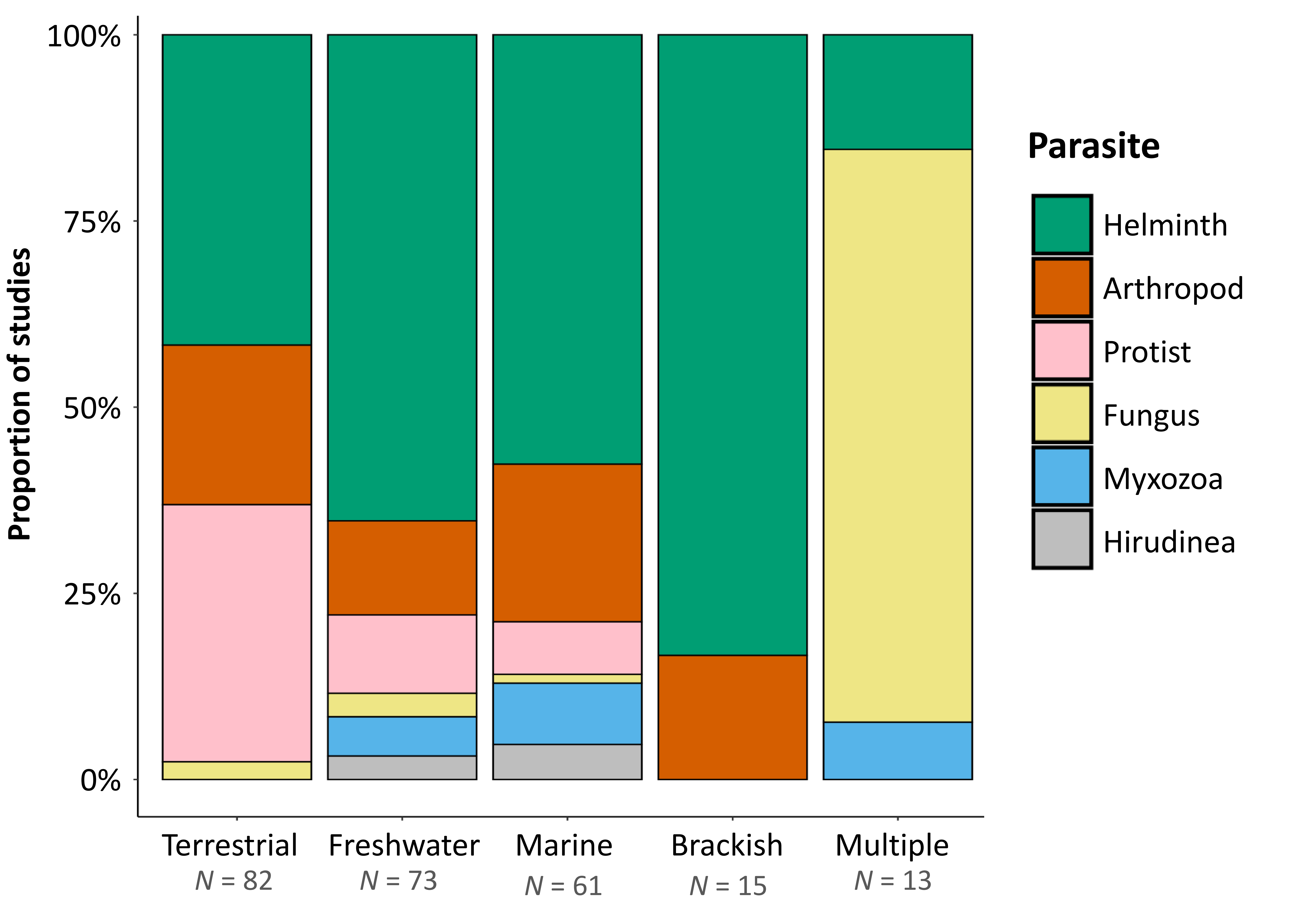
Fig. S9.** Distribution of parasite types among type of environment studied.

**
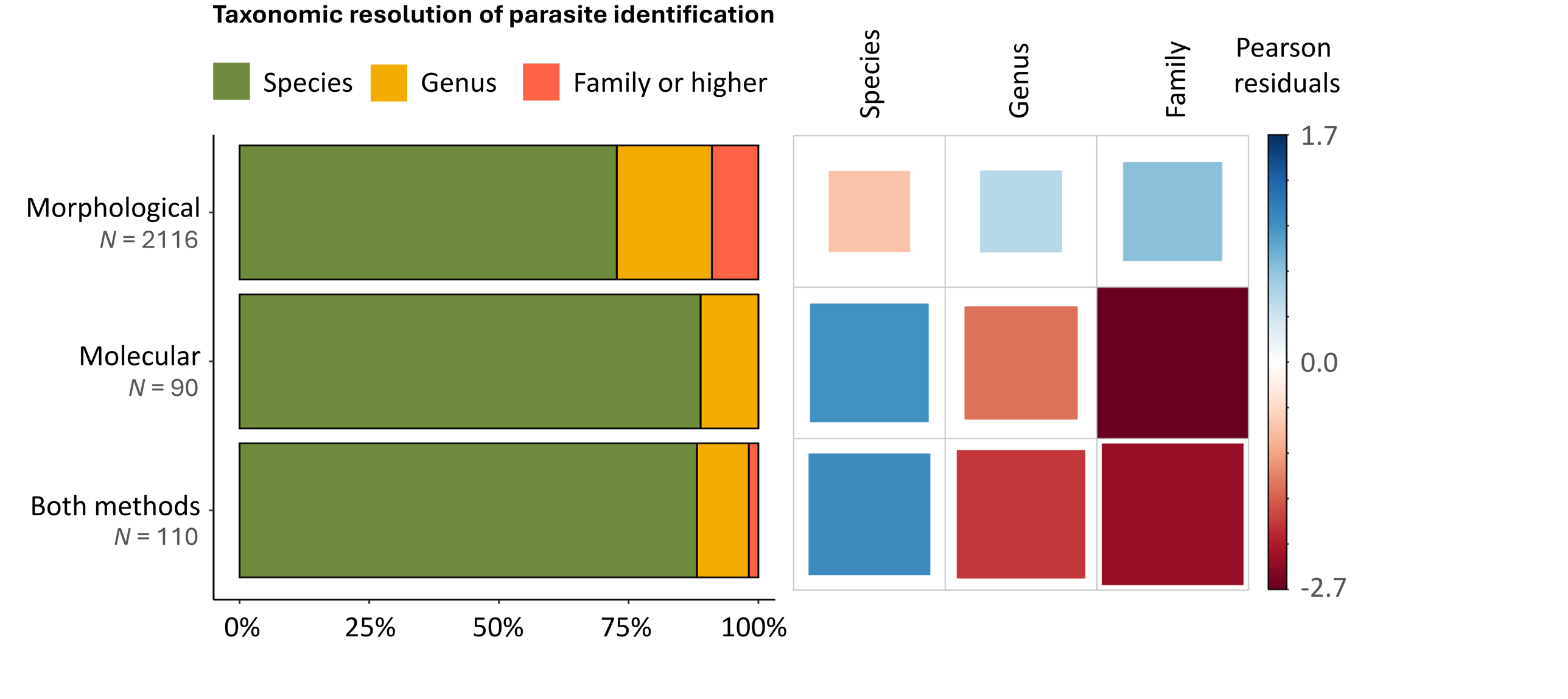
Fig. S10.** Taxonomic resolution of parasite identification depending on the broad identification method. Sample size values refer to number of identified parasites across studies. The plot matrix on the right shows, using the Pearson residuals of the Chi-squared test, that the specificity of parasite identification tends to be higher when molecular tools are used.

**
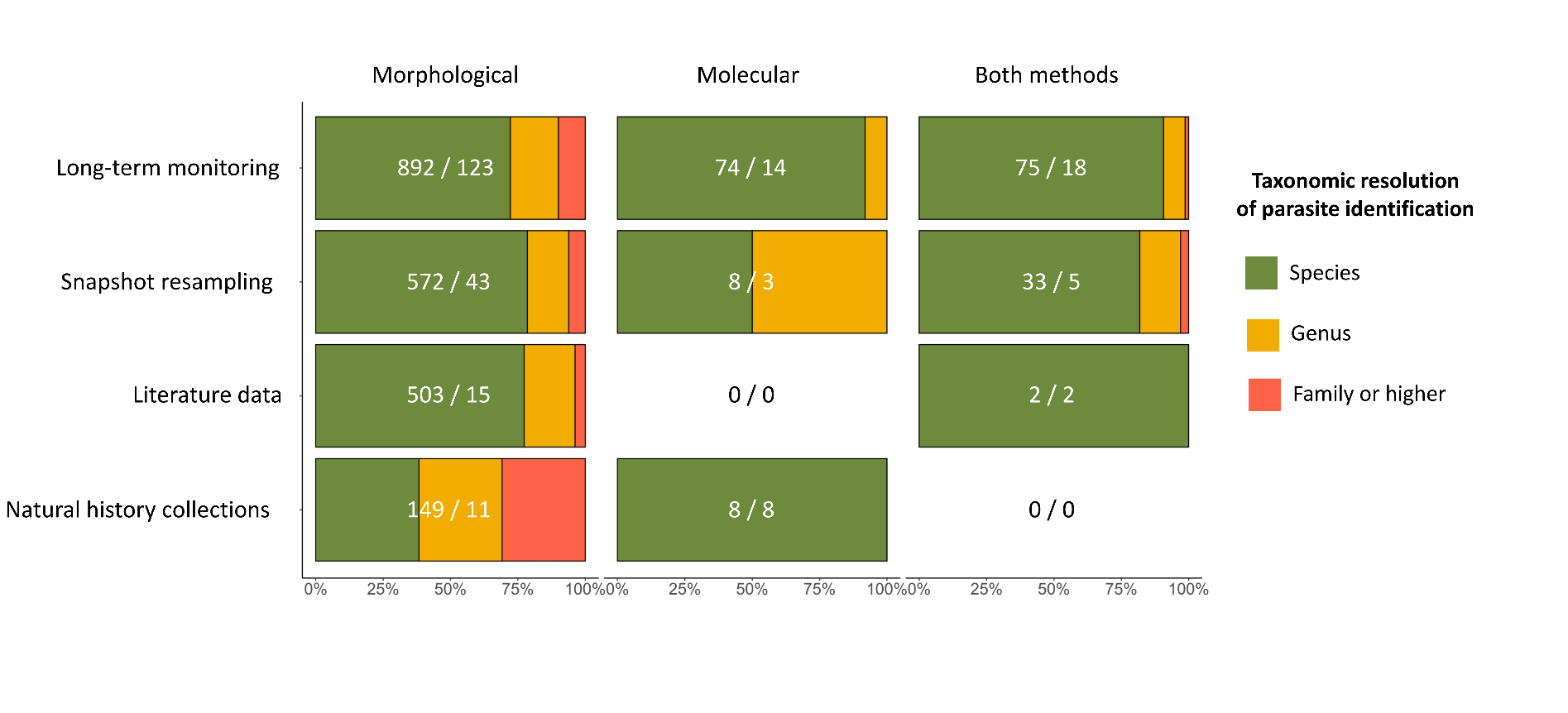
Fig. S11.** Summary of the taxonomic resolution of parasite identification depending on both research approach and identification method. Values on the bars indicate the number of parasite taxa included in the category on the left, and the corresponding number of studies on the right.
